# The effects of combined amplitude and high-frequency vibration on physically inactive osteopenic postmenopausal women

**DOI:** 10.3389/fphys.2022.952140

**Published:** 2022-09-07

**Authors:** Peter Fernandez, Marion Pasqualini, Hervé Locrelle, Myriam Normand, Christine Bonneau, Marie-Hélène Lafage Proust, Hubert Marotte, Thierry Thomas, Laurence Vico

**Affiliations:** ^1^ SAINBIOSE, U1059, Laboratory of Osteoarticular Tissue Biology, INSERM, University of Lyon, Saint-Etienne, France; ^2^ Rheumatology Department, University Hospital of Saint-Etienne, Saint-Etienne, France; ^3^ Biology and Pathology Department, University Hospital of Saint-Etienne, Saint-Etienne, France

**Keywords:** whole-body vibration, age-related bone loss, fracture risk, dose-response, postmenopausal women

## Abstract

**Purpose:** To evaluate whole-body vibration (WBV) osteogenic potential in physically inactive postmenopausal women using high-frequency and combined amplitude stimuli.

**Methods:** Two-hundred fifty-five physically inactive postmenopausal women (55–75 years) with 10-year major osteoporotic fracture risk (3%–35%) participated in this 18-month study. For the first 12 months, the vibration group experienced progressive 20-min WBV sessions (up to 3 sessions/week) with rest periods (30–60 s) between exercises. Frequencies (30–50 Hz), with low (0.2–0.4 mm) and high (0.6–0.8 mm) amplitude stimuli were delivered *via* PowerPlate Pro5 platforms producing accelerations of (0.75–7.04 g). The last 6 months for the treatment group were a follow-up period similar to control. Serum bone remodelling markers [C-terminal crosslinked telopeptide of type-1 collagen (CTX), procollagen type-1 N-terminal propeptide (P1NP), bone alkaline phosphatase (BAP) and sclerostin] were measured at fasting. CTX and P1NP were determined by automated chemiluminescence immunoassay, bone alkaline phosphatase (BAP) by automated spectrophotometric immunoassay, and sclerostin by an enzyme-immunoassay. Bone mineral density (BMD) of the whole-body, proximal femur and lumbar vertebrae was measured by dual-energy X-ray absorptiometry (DXA). Bone microarchitecture of the distal non-dominant radius and tibia was measured by high-resolution peripheral quantitative computed tomography (HR-pQCT).

**Results:** Femoral neck (*p* = 0.520) and spine BMD (*p* = 0.444) failed to improve after 12 months of WBV. Bone macro and microstructural parameters were not impacted by WBV, as well as estimated failure load at the distal radius (*p* = 0.354) and tibia (*p* = 0.813). As expected, most DXA and HR-pQCT parameters displayed age-related degradation in this postmenopausal population. BAP and CTX increased over time in both groups, with CTX more marginally elevated in the vibration group when comparing baseline changes to month-12 (480.80 pmol/L; *p* = 0.039) and month-18 (492.78 pmol/L; *p* = 0.075). However, no differences were found when comparing group concentrations only at month-12 (506.35 pmol/L; *p* = 0.415) and month-18 (518.33 pmol/L; *p* = 0.480), indicating differences below the threshold of clinical significance. Overall, HR-pQCT, DXA bone parameters and bone turnover markers remained unaffected.

**Conclusion:** Combined amplitude and high-frequency training for one year had no ameliorating effect on DXA and HR-pQCT bone parameters in physically inactive postmenopausal women. Serum analysis did not display any significant improvement in formation and resorption markers and also failed to alter sclerostin concentrations between groups.

## Introduction

Osteoporosis affects skeletal integrity, whereby bone mineral density (BMD) is decreased in addition to microarchitectural deterioration (Kanis et al., 2019). This results in an increased risk of fractures impacting mobility (Kanis et al., 2019) and quality of life (Rhodes et al., 2000), with repercussions at an individual and societal level worldwide (Rashki Kemmak et al., 2020; Salari et al., 2021). In Europe, fracture rates and treatment costs are projected to surge by 25% (Willers et al., 2022). Recent European statistics have also demonstrated a stark contrast in the numbers of osteoporotic cases between males and females, with females affected nearly four times as much (Willers et al., 2022). This increased risk in women is largely attributed to the effect of menopause (The ESHRE Capri Workshop Group, 2010), and despite advances in prevention, screening and management of this condition, osteoporosis continues to remain a significant challenge worldwide (Salari et al., 2021; Willers et al., 2022). For this reason, females are the primary focus of this study.

Several pharmacological treatments and exercise interventions are relied upon to manage osteoporosis (Beck, 2022; Palacios, 2022). However, the difficulty with medication is finding the appropriate balance between evidence-based medicine and proven treatment strategies (Ragucci and Shrader, 2011; Gregson et al., 2022). This is further complicated when integrating suitable exercise interventions for the management of this condition. Previous studies have highlighted the effectiveness of exercise in the management of osteoporosis (Gupta and March, 2016; Koshy et al., 2022). However, significant consideration is required since exercise can substantially increase fall risk and needs to be adapted to the individual (Gupta and March, 2016; Benedetti et al., 2018). Furthermore, prevention with physical activity interventions has not always yielded positive effects (Rhodes et al., 2000; Borer, 2005; Ma et al., 2013). As a result, modalities such as whole-body vibration (WBV) were proposed to harness the potential of physical activity by combining simplicity, ease of administration and encouraging adherence. Since WBV can be easily adapted into an everyday routine, it has garnered a lot of attention over the years as a potential treatment modality for osteoporosis prevention, particularly in postmenopausal women (Marin-Puyalto et al., 2018). WBV involves the transmission of mechanical stimuli delivered *via* different vibration platforms (vertical, rotational, or lateral planes) that transfer forces to skeletal segments like other forms of exercise (Marin-Puyalto et al., 2018). The effects of WBV have been extensively investigated (Verschuren et al., 2004; Gusi et al., 2006; Von Stengel et al., 2011a; Von Stengel et al., 2011b; Slatkovska et al., 2011; Verschueren et al., 2011; Lai et al., 2013; Stolzenberg et al., 2013; Slatkovska et al., 2014; Liphardt et al., 2015; de Oliveira et al., 2019) resulting in disparities between studies.

Following seminal studies by Rubin et al. (2004) to evidence the impact of WBV on bone, abundant studies ranging from short to long-duration exposure to WBV have been tested (Gómez-Cabello et al., 2014; Kiel et al., 2015). In the 11-week study published by Gomez-Cabello et al. (2014), bone parameters in a relatively small population were examined using high-amplitude and high-frequency vibration stimuli. Particular focus was on dual-energy X-ray absorptiometry (DXA) derived parameters at the femoral neck and lumbar spine along with added peripheral quantitative computer tomography (pQCT) parameters examining the cortical and trabecular BMD of the radius and tibia. Despite this combination, no conclusive effects on bone were identified. Similarly, studies 6-month in length showed parallels in signal characteristics observed in the 11-week study; however, muscle strength and hip density were also incorporated into the protocol (Verschueren et al., 2004; Verschueren et al., 2011).

Interestingly, two studies of the same 6-month duration explored effects on bone turnover markers (cross-linked C-telopeptide of type I collagen and osteocalcin) alongside DXA outcomes using similar high-frequency and high-amplitude stimuli. Both reached similar conclusions with varying bone and serum results despite utilising similar vibration platforms (Verschuren et al., 2004; Sen et al., 2020). Some studies of much longer duration (12 months or greater) have shifted the focus more towards incorporating advancements in 3D imaging modalities, moving away from the reliance on traditional 2D DXA parameters to draw more in-depth conclusions associated with longitudinal changes in bone (Slatkovska et al., 2011). Studies have experimented with a range of frequencies (Slatkovska et al., 2011) and exposure intervals (Liphardt et al., 2015), using High Resolution Peripheral Computer Tomography (HR-pQCT) (Slatkovska et al., 2011; Liphardt et al., 2015) or novel Magnetic Resonance Imagining (MRI) techniques (Rajapakse et al., 2021) to examine the effects of WBV on bone geometry and microstructural parameters. However, the extensive focus on 3D imaging modalities has exacerbated differences of opinion regarding the efficacy of WBV on bone.

These studies alone highlight several gaps in the literature. Since the osteogenic response to physical activity reduces with age (Rubin et al., 1992), alterations in the length and frequency of sessions could be necessary (Gómez-Cabello et al., 2014). Weekly sessions have varied between one and three, with some varying the training intensity by modifying the signal characteristics and rest periods (Beck and Norling, 2010; Slatkovska et al., 2011; Sen et al., 2020). Despite modifications to signal attributes seen in studies to date, the role of amplitude in WBV bone response is still in question. Physical activity is known to produce a range of amplitudes depending on the type of exercise performed (Deere et al., 2016). Since WBV is a substitute for physical activity, it is essential to replicate this aspect as closely as possible. Frequency variations (high/low), varying amplitudes (<1 g/> 1 g), as well as exposure intervals to the vibration stimuli, have significantly differed between studies and are critical elements of any vibration signal (Prisby et al., 2008). The lack of studies investigating combined amplitude creates a new research opportunity. Physical activity levels prior to intervention were also alluded to as a possible explanation for the lack of observable effect (Rajapakse et al., 2021), which has not been a consideration in studies thus far. Accounting for this might reveal a relationship between WBV and osteogenic potential. Finally, several studies have already explored the response of formation and resorption of bone markers but have not investigated sclerostin, which is known to increase with age and fluctuate based on activity levels (Robling et al., 2008; Amrein et al., 2012). The inclusion of this could capture the subtle responses to combined amplitude stimuli.

In light of the differences outlined above, this study aimed to investigate the role of high-frequency and combined amplitude stimuli over 12 months on physically inactive postmenopausal women. The primary research objective was to investigate changes specific to the femoral neck, while the secondary objective was to measure bone geometry and microarchitectural changes when exposed to such stimuli. In addition, a 6-month follow-up after vibration training was added to observe any sustained osteogenic benefits. It was hypothesised that bone parameters would be improved, with positive osteogenic effects sustained following WBV therapy.

## Materials and methods

### Ethical approval

Written informed consent for all experiment protocols was obtained from each participant and conformed to the standards set by the latest revision of the Declaration of Helsinki and the committee of human rights protection, southeast, France (N°0908095). ClinicalTrials.gov Identifier (NCT01982214).

### Participant information and study design

**FIGURE 1:**
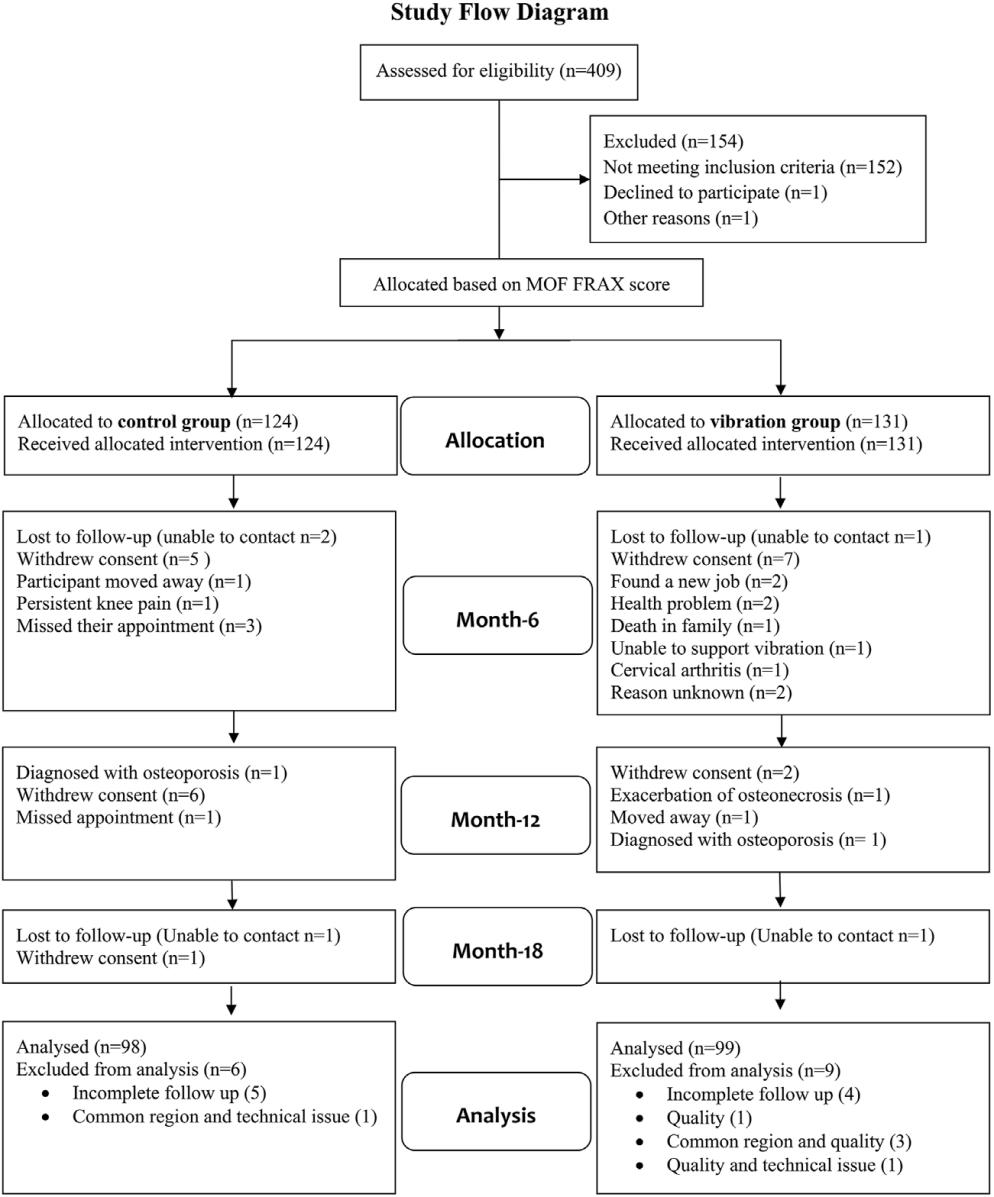
Study flow chart.

**FIGURE 2:**
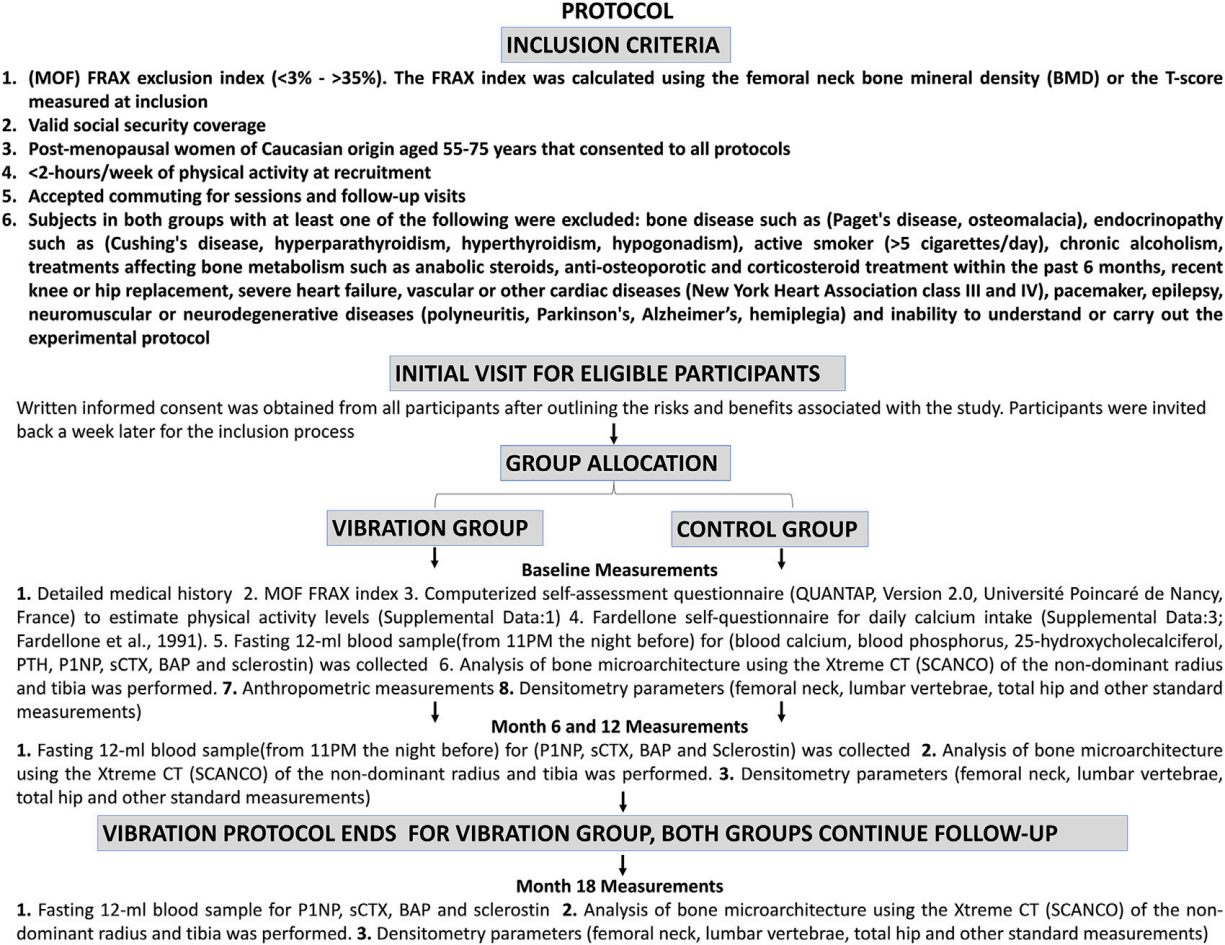
Protocol flow chart.

255 sedentary (< 2 h of physical activity/week at recruitment) postmenopausal women (55–75 years) with absent menses > 1 year, participated in this 18-month non-randomised clinical trial (Supplementary Material S1; Figures 1, 2). Physical activity levels were screened using a questionnaire and further estimated by a computerized self-administered self-assessment questionnaire (QUANTAP, Version 2.0, Université Poincaré de Nancy, France) to obtain an overview of the participants’ activity levels at recruitment (Supplemental Material S1). The first 12 months consisted of the vibration protocol (vibration group) and regular visits (control group). The 6 months thereafter was a follow-up period for both groups. Since difficulties with randomisation were anticipated, participants were given the choice to select their preferred group allocation for the duration of the study. Potential bias resulting from this was effectively addressed since participants were matched according to their FRAX scores for major osteoporotic fracture (MOF) 10 year absolute risk ranging from (3%–35%) to ensure group comparability.

### Training program

Only the vibration group carried out this protocol. All participants familiarised themselves with the vibration protocol and equipment beforehand. Twenty minute sessions of light squatting and stretching exercises (max 3 sessions/week, up to 130 sessions total) during the 12-month vibration protocol were performed and recorded in the participants’ logbook. These exercises were for the sole purpose of maintaining the participants’ motivation given the duration of the vibration training sessions. The first session was shorter and interspersed with rest periods, and a fitness instructor constantly ensured the participants’ safety.

The vibration characteristics were: frequency (30–50 Hz), with a combination of low-amplitude (0.2–0.4 mm) and high-amplitude (0.6–0.8 mm) stimuli were delivered using PowerPlate Pro5 airdaptive system (Performance Health Systems, LLC, NorthBrook, IL, United States) to generate an acceleration profile of (0.75–7.04 g). This system delivered vibration stimuli tri-axially (X,Y,Z axis) and possessed a self-adjusting air cushion system that distributed participants’ weight across the platform to mitigate against signal dampening. The chosen vibration amplitudes and frequencies were applicable to elderly people to obtain an acceleration close to 3 g, reproduced by fast walking or osteogenic sports (Vainionpää et al., 2006). Initially, all participants started with low-amplitude stimuli; however, exercise repetition, vibration frequency and amplitude progressively changed throughout the study (Supplementary Material S2). All participants were requested not to modify their regular activity levels, avoiding impact activities/sports throughout the entire study.

### Biochemistry

All samples were obtained in the morning following an overnight fast. Serum concentrations of C-terminal crosslinked telopeptide of type-1 collagen (CTX, pmol/L) and procollagen type-1 N-terminal propeptide (P1NP, µg/L) were determined by automated chemiluminescence immunoassay (IDS-iSYS automated analyser, Boldon, United Kingdom), while bone alkaline phosphatase (BAP, µg/L) was measured using an automated spectrophotometric immunoassay (IDS-iSYS automated analyser, Boldon, United Kingdom). Intra-assay CV were < 4.9%, < 3.0%, and < 2%, while inter-assay CV were < 8.8%, < 5.3, and < 9% for CTX, P1NP and BAP respectively. An enzyme-immunoassay (EIA) kit was used to quantify sclerostin levels (SOST, ng/ml) (Quidel Corporation San Diego, CA, United States) with precision CV’s of 3.7%–4.2% and 4.3%–4.8% for within-run and between run respectively.

### Dual-energy X-ray absorptiometry

Fat, lean body and total body mass in grams, along with standard BMD measurements at the femoral neck (g/cm^2^), total hip (g/cm^2^), and lumbar spine (L1-L4) (g/cm^2^), were measured using DXA (GE, Lunar iDXA, Milwaukee, WI, United States). Additional femoral neck analyses were performed using 3D-Shaper (Galgo Medical, Barcelona, Spain) to obtain cortical vBMD (mg/cm^3^) and trabecular vBMD (mg/cm^3^) of the femur and femoral neck. Lumbar spine trabecular bone score (g/cm^2^) was attained using TBS iNsight (Medimaps, Geneva, Switzerland). All measurements were performed by the same qualified technician according to manufacturer’s guidelines.

### High-resolution peripheral quantitative computed tomography measurements

All microarchitectural bone measurements were obtained using HR-pQCT (Scanco Medical, Bassersdorf, Switzerland) of the non-dominant radius and tibia unless a fracture was reported in the region of interest. Scans were performed by the same qualified operator by positioning the reference line at the endplate of the radius and tibia on an anteroposterior scout view. Using this line, the standard scan region of interest was found automatically with the first slice 9.5 mm and 22.5 mm down to the reference line for the distal radius and tibia respectively. The following settings were used: peak energy, 60 kVp; X-ray tube current, 900 mA; matrix size, 1,536 × 1,536.A slack of 110 CT slices were acquired over a 9 mm length consisting of 110 CT slices with an isotropic voxel size of 82 µm, with an effective dose of 3 µSv in approximately 3 min. Image quality was scored ranging from grade 1 (highest quality) to grade 5 (unacceptable), using manufacturers’ recommendations (Scanco Medical). A preliminary quality grading was made prior to image acquisition, and repeated measurements were made for all scans with insufficient quality (grade 4 or 5). Thus, only scans with quality grade 1–3 (none, minor or moderate motion artefacts) were used for subsequent image analysis. After manual correction of the periosteal and endosteal contours when needed, total vBMD was calculated as the total amount of mineral divided by the total bone volume within the periosteal contour. This volume was then separated into cortical and trabecular compartments, each compartment being analysed separately. Images were analysed using the advanced cortical evaluation protocol provided by the manufacturer (Burghardt et al., 2010).

The following parameters were measured for the radius and tibia and analysed using Image Processing Language (IPL;version-6, Scanco Medical): total BMD (mg HA/cm^3^), trabecular bone volume fraction (BV/TV, %), trabecular inhomogeneity (Tb.1/N.SD; mm), trabecular vBMD (mg HA/cm^3^), cortical vBMD (mg HA/cm^3^), cortical area (Ct.Ar; mm), cortical thickness (Ct.Th; mm), cortical porosity (Ct.Po; %) and cortical perimeter (Ct.Pm; mm). Finite element analysis using the advanced evaluation script (version 1.0, Scanco Medical) evaluated failure load (FL; kN), compartment load distribution (Load_trab.prox,_ Load_trab.dist;_ %) and average Von Mises stress (Stress_VM_; Mpa) of the cortical and trabecular compartments. The coefficient of variation has been established elsewhere (Vico et al., 2008). The reproducibility of HR-pQCT density measurements ranged from 0.5% to 1.1%. In comparison, reproducibility of the structural parameters (number of trabeculae, trabecular thickness, and cortical thickness) was slightly lower, with coefficients ranging from 0.7% to 4.5%. The reproducibility of the measurement was similar at the distal radius and the distal tibia. Finally, total volume (mm^3^) and muscle volume fraction (MV/TV, %) of the distal tibia was performed using soft tissue analysis script provided by Scanco Medical (Erlandson et al., 2017).

### Statistical analysis

To estimate the effect size, the DXA BMD at the femoral neck was used. An analysis of four cohort studies (Arlot et al., 1997; Pedrazzoni et al., 2003; Gjesdal et al., 2004; Emaus et al., 2006) evaluating average bone loss in postmenopausal women aged between 55 and 75 years estimated the change in BMD over 1 year and found that a loss of 1% per year was observed. Another study evaluating the effects of walking on BMD in postmenopausal women (Martyn-St James and Carroll, 2008) reported an effect of ^+^2% compared to controls. Additionally, another study comparing the effects of walking with those of WBV (Gusi et al., 2006) observed a ^+^2% difference in WBV from walking on BMD. Thus, we wished to demonstrate an effect of ^+^4% with the WBV compared to the control group (a Delta of 0.04). Considering this desired delta, with a standard deviation of 0.10, a power of 80% and an alpha of 5%, the number of subjects required would consist of 100 participants in each group. A total of 240 women was expected to be included, 120 women per group, taking into account those who would be lost at follow-up.

An independent samples *t*-test was used to assess differences in baseline characteristics and for the selection of appropriate covariates. All quantities were analysed in STATA-17 (StataCorp LP, College Station, Texas, United States) using a linear mixed model with a random intercept and robust standard errors. This model was selected given the multiple timepoints associated with each participant and the ability to handle missing values. Fixed factors were group (2 levels: control, vibration) and month (4 levels: 0,6,12,18), while the random factor was participants (*n* = 197). Age was treated as a continuous factor. Overall model fit and normality was assessed using quartile-quartile plots of the residuals. All dependant variables during the vibration phase were analysed in the following way: (Baseline vs. month-6 and Baseline vs. month-12). Baseline compared with follow-up used: (Baseline vs. month-18). Finally, to analyse the prolonged effect of vibration (Month-12 vs. Month-18) was compared. Bonferroni correction was applied for all comparisons with results presented as contrasts between groups, and the significance level was set to *p* < 0.05.

## Results

This non-randomised clinical trial matched participants according to their FRAX scores for major osteoporotic fracture (MOF) 10 years-absolute risk ranging from (3%–35%) to ensure group comparability. Statistical analysis was performed using 197 participants (Figure 1) having four timepoints (baseline, 6, 12 and 18 months). For all 197 participants, HR-pQCT image quality and common region were (≤ 3) and (≥ 70%) respectively (Table 1; Figure 1). Participants’ baseline characteristics can be found in (Tables 2, 3). Vibration protocol adherence was good with 110.02 ± 14.25 (mean ± SD) vibration sessions from a maximum of 130 completed by the vibration group. All within groups differences are presented in (Tables 4 –7) and significant between group differences are presented in (Table 8).

**TABLE 1:** Breakdown of the total number of participants analysed for HR-pQCT parameters, based on having 4-time points, image quality (≤ 3) and common region (≥ 70%). Total number of participants (*n* = 197): Control (*n* = 98), Vibration (*n* = 99). Common region for tibia (mean ± SD): Control (89.05 ± 7.27) Vibration (88.05 ± 9.06). Common region for radius (mean ± SD): Control (82.00 ± 10.64) Vibration (82.70 ± 12.41).

Total number of participants analysed for HR-pQCT parameters					
Group					
Control			Vibration		
Radius only	Tibia only	Radius and Tibia	Radius only	Tibia only	Radius and Tibia
10	33	55	9	26	64

**TABLE 2:** Participants’ characteristics and DXA parameters at baseline (See Supplementary Material S3) and (Fardellone et al., 1991) for Fardellone self-questionnaire. (See Supplementary Material S1) for lifetime activity questionnaire (QUANTAP). QUANTAP total lifetime activity and current level of physical activity at recruitment were reported in Kilojoules/Kilograms (Kj/Kg).

Group					
	Control (*n* = 98)		Vibration (*n* = 99)		*p*-value
	Mean	SD	Mean	SD	
*Participants*’ *baseline characteristics*					
Age	66.1	5.39	63.27	4.94	< 0.001
(MOF) Frax score	6.04	2.63	5.41	2.09	0.062
(Fardelonne) daily calcium intake (mg)	762.24	383.92	677.9	323.02	0.099
(QUANTAP) total lifetime activity at recruitment (Kj/kg)	108,8338.88	625,610.81	112,2443.84	579,877.59	0.698
(QUANTAP) current physical activity levels at recruitment (Kj/kg)	22,965.06	16,743.94	24,198.09	15,973.68	0.607
Calcium (mmol/L)	2.29	0.25	2.28	0.08	0.739
Phosphate (mmol/L)	1.21	0.19	1.21	0.18	0.983
25-OH Vitamin D (µg)	27.04	10.91	29.73	9.82	0.07
Parathyroid hormone (ng/L)	29.94	11.35	29.77	10.44	0.911
Height (cm)	159.33	5.82	158.12	5.43	0.133
Body mass (kg)	66.8	13.15	63.5	10.71	0.058
BMI (kg/m^2^)	26.21	4.84	25.11	4.14	0.089
*DXA and * *3D-* *Shaper*					
Total BMD (g/cm^2^)	1.02	0.09	1.01	0.1	0.371
Total T-score (SD)	0.05	0.93	−0.08	0.98	0.371
Fat mass (g)	26,845.52	9,543.16	24,673.83	7,314.66	0.074
Lean mass (g)	37,858.96	4,363.89	36,812.57	4,169.27	0.087
Body mass (g)	66,806	13,157.84	63,545.19	10,713.35	0.058
BMD spine L1-L4 (g/cm^2^)	1.06	0.16	1.04	0.15	0.398
T-score spine L1-L4 (SD)	−0.86	1.35	−1.01	1.21	0.398
Trabecular bone score L1-L4 (g/cm^2^)	1.26	0.09	1.28	0.1	0.162
BMD femoral neck (g/cm^2^)	0.85	0.09	0.84	0.09	0.253
T-score femoral neck (SD)	−1.07	0.73	−1.2	0.79	0.253
BMD Total Hip (g/cm^2^)	0.89	0.1	0.87	0.11	0.071
T-score total hip (SD)	−0.88	0.85	−1.11	0.88	0.071
3D-Shaper total cortical vBMD (mg/cm^3^)	803.9	84.05	788.19	84.62	0.193
3D-Shaper total trabecular vBMD (mg/cm^3^)	140.42	33.09	135.05	35.21	0.272
3D-Shaper cortical vBMD of the neck (mg/cm^3^)	803.7	74.97	786.53	74.77	0.109
3D-Shaper trabecular vBMD of the neck (mg/cm^3^)	187.91	37.06	185.99	37.51	0.719

**TABLE 3:** HR-pQCT (Radius and Tibia) and serum parameters at baseline.

Group					
	Control (*n* = 98)		Vibration (*n* = 99)		*p*-value
	Mean	SD	Mean	SD	
*HR-pQCT (Tibia)*					
Total BMD (mgHA/cm^3^)	257.5	49.15	253.4	48.44	0.576
Trabecular vBMD (mgHA/cm^3^)	154.53	34.96	152.4	35.93	0.688
BV/TV (%)	0.13	0.03	0.13	0.03	0.686
Trabecular inhomogeneity (Tb.1/N.SD; mm)	0.29	0.16	0.35	0.38	0.157
Cortical area (mm^2^)	96.6	14.51	92.39	15.72	0.065
Cortical vBMD (mgHA/cm^3^)	795.6	60.3	806.31	64.9	0.256
Cortical perimeter (mm)	99.49	7.38	99.86	6.7	0.728
Cortical thickness (mm)	1.07	0.18	1.02	0.18	0.091
Cortical porosity (%)	0.08	0.03	0.07	0.03	0.051
Ultimate load (N)	−8028.69	1,265.94	−8020.71	1,320.72	0.967
Trabecular load vs. whole bone load (distal)	0.52	0.09	0.53	0.1	0.397
Trabecular load vs. whole bone load (proximal)	0.32	0.08	0.33	0.09	0.32
Trabecular Von Mises stress (MPa)	52.76	7.11	54.06	7.18	0.225
Cortical Von Mises stress (MPa)	86.01	2.21	86.19	2.41	0.612
Total Muscle Volume (mm^3^)	19,625.05	4,851.78	18,770.96	3,965.69	0.201
MV/TV	0.55	0.15	0.57	0.13	0.453
*HR-pQCT (Radius)*					
Total BMD (mgHA/cm^3^)	295.95	67.08	295.05	59.35	0.934
Trabecular vBMD (mgHA/cm^3^)	149.62	39.62	142.75	41.17	0.321
BV/TV (%)	0.12	0.03	0.12	0.03	0.319
Trabecular inhomogeneity (Tb.1/N.SD; mm)	0.29	0.21	0.33	0.22	0.368
Cortical area (mm^2^)	48.88	8.26	48.73	7.64	0.915
Cortical vBMD (mgHA/cm^3^)	847	56.1	863.29	59.37	0.101
Cortical perimeter (mm)	68.8	10.67	66.76	5.04	0.145
Cortical thickness (mm)	0.81	0.16	0.82	0.15	0.748
Cortical porosity (%)	0.025	0.013	0.02	0.009	0.011
Ultimate load (N)	−2,859.75	551.01	−2901.77	541.36	0.654
Trabecular load vs. whole bone load (distal)	0.44	0.07	0.44	0.1	0.952
Trabecular load vs. whole bone load (proximal)	0.16	0.06	0.16	0.06	0.656
Trabecular Von Mises stress (MPa)	42.42	6.29	43.61	7.24	0.31
Cortical Von Mises stress (MPa)	78.92	3.81	79.99	3.55	0.092
*Serum*					
P1NP (µg/L)	55.77	23.87	56.17	18.39	0.897
CTX (pmol/L)	4,141.05	2,379.15	4,332.82	1,836.07	0.527
BAP (µg/L)	12.25	4.12	11.71	3.6	0.334
Sclerostin (ng/ml)	0.64	0.15	0.6	0.15	0.062

**TABLE 4:** Summary of all DXA and 3D-Shaper parameters.

	Control					Vibration						
	Contrast	% change	*p*-value	95% lower bound	95% upper bound	Contrast	% change	*p*-value	95% lower bound	95% upper bound	*p*-value group*month	*p*-value month
Total BMD (g/cm^2^)											*p* = 0.0980	*p*<0.001
Month 6 vs. Baseline	−0.005	−0.51	0.00	−0.008	−0.002	−0.004	−0.36	0.01	−0.007	−0.001		
Month 12 vs. Baseline	−0.011	−1.07	0.00	−0.014	−0.008	−0.007	−0.72	0.00	−0.011	−0.004		
Month 18 vs. Baseline	−0.011	−1.08	0.00	−0.016	−0.006	−0.010	−1.02	0.00	−0.014	−0.007		
Fat mass (g)											*p* = 0.0107	*p* = 0.262
Month 6 vs. Baseline	368.804	1.37	0.32	−135.817	873.425	56.752	0.23	1.00	−333.840	447.344		
Month 12 vs. Baseline	947.120	3.53	0.00	253.847	1640.393	170.848	0.69	1.00	-298.648	640.345		
Month 18 vs. Baseline	616.845	2.30	0.11	−70.477	1304.166	554.351	2.25	0.03	25.956	1082.746		
Lean mass (g)											*p* = 0.0268	*p* < 0.001
Month 6 vs. Baseline	522.864	1.38	0.00	185.875	859.852	168.943	0.46	0.98	−150.549	488.434		
Month 12 vs. Baseline	878.791	2.32	0.00	520.487	1237.095	359.627	0.98	0.01	71.778	647.476		
Month 18 vs. Baseline	759.616	2.01	0.00	409.126	1110.106	476.753	1.30	0.00	132.296	821.210		
Body mass (g)											*p* = 0.0015	*p* = 0.005
Month 6 vs. Baseline	885.310	1.33	0.00	211.027	1559.593	222.018	0.35	1.00	−310.917	754.954		
Month 12 vs. Baseline	1808.351	2.71	0.00	952.759	2663.943	522.380	0.82	0.10	−57.210	1101.970		
Month 18 vs. Baseline	1350.420	2.02	0.00	521.401	2179.439	1014.991	1.60	0.00	398.553	1631.429		
BMD spine (g/cm^2^)											*p* = 0.4447	*p* < 0.001
Month 6 vs. Baseline	−0.007	−0.64	0.21	−0.015	0.002	−0.007	−0.68	0.04	−0.014	0.000		
Month 12 vs. Baseline	−0.011	−1.04	0.00	−0.020	−0.002	−0.012	−1.18	0.00	−0.020	−0.005		
Month 18 vs. Baseline	−0.007	−0.62	0.35	−0.016	0.003	−0.014	−1.33	0.00	−0.022	−0.005		
Trabecular bone score L1-L4 (g/cm^2^)											*p* = 0.5035	*p* < 0.001
Month 6 vs. Baseline	0.005	0.41	1.00	−0.008	0.019	0.010	0.75	0.41	−0.004	0.023		
Month 12 vs. Baseline	−0.004	−0.33	1.00	−0.017	0.009	−0.005	−0.37	1.00	−0.017	0.007		
Month 18 vs. Baseline	−0.005	−0.37	1.00	−0.019	0.009	−0.014	−1.13	0.02	−0.028	−0.001		
BMD femoral neck (g/cm^2^)											*p* = 0.5201	*p* = 0.180
Month 6 vs. Baseline	0.002	0.24	1.00	-0.004	0.008	−0.004	−0.43	0.46	−0.009	0.002		
Month 12 vs. Baseline	−0.001	−0.13	1.00	−0.006	0.004	−0.004	−0.51	0.19	−0.010	0.001		
Month 18 vs. Baseline	−0.003	−0.33	1.00	−0.010	0.004	−0.008	−0.98	0.01	−0.015	−0.002		
BMD Total Hip (g/cm^2^)											*p* = 0.4923	*p* < 0.001
Month 6 vs. Baseline	−0.003	−0.39	0.17	−0.008	0.001	−0.002	−0.19	1.00	−0.005	0.002		
Month 12 vs. Baseline	−0.006	−0.72	0.00	−0.011	−0.002	−0.003	−0.38	0.16	−0.007	0.001		
Month 18 vs. Baseline	−0.008	−0.89	0.00	−0.013	−0.003	−0.008	−0.90	0.00	−0.012	−0.003		
3D-Shaper total cortical vBMD (mg/cm^3^)											*p* = 0.3041	*p* = 0.001
Month 6 vs. Baseline	−3.247	−0.40	1.00	−9.769	3.274	1.349	−0.17	1.00	−7.985	5.287		
Month 12 vs. Baseline	−3.129	−0.39	1.00	−9.308	3.051	1.149	0.15	1.00	−5.052	7.351		
Month 18 vs. Baseline	−5.710	−0.71	0.15	−12.409	0.990	−6.931	−0.88	0.08	14.370	0.509		
3D-Shaper total trabecular vBMD (mg/cm^3^)											*p* = 0.0807	*p* < 0.001
Month 6 vs. Baseline	−1.142	−0.81	0.35	−2.732	0.447	0.825	0.61	1.00	−1.209	2.859		
Month 12 vs. Baseline	−2.707	−1.93	0.00	−4.621	−0.794	−0.470	−0.35	1.00	−2.387	1.448		
Month 18 vs. Baseline	−3.669	−2.61	0.00	−5.986	−1.353	−2.925	−2.17	0.05	−5.813	−0.038		
3D-Shaper cortical vBMD of the neck (mg/cm^3^)											*p* = 0.9032	*p* = 0.005
Month 6 vs. Baseline	−1.555	−0.19	1.00	−7.752	4.641	−1.425	−0.18	1.00	−8.257	5.407		
Month 12 vs. Baseline	−0.416	−0.05	1.00	−6.388	5.556	1.138	0.14	1.00	−4.901	7.176		
Month 18 vs. Baseline	−4.495	−0.56	0.27	−10.396	1.407	−5.740	−0.73	0.24	−13.094	1.614		
3D-Shaper trabecular vBMD of the neck (mg/cm^3^)											*p* = 0.3418	*p* = 0.030
Month 6 vs. Baseline	−0.731	−0.39	1.00	−2.915	1.452	0.328	0.18	1.00	−2.218	2.875		
Month 12 vs. Baseline	−2.875	−1.53	0.18	−6.384	0.635	−0.951	−0.51	1.00	−3.218	1.316		
Month 18 vs. Baseline	−2.969	−1.58	0.11	−6.271	0.334	−3.791	−2.04	0.02	−7.260	−0.323		

### Dual-energy X-ray absorptiometry and 3D-Shaper

All DXA and 3D-Shaper parameters are presented in (Table 4). Other than a similar and continued decrease over time in all participants, no significant improvement in BMD of the femoral neck, total hip, total cortical and trabecular vBMD of the femur, cortical and trabecular vBMD at the femoral neck, total BMD, bone mass, and BMD and trabecular bone score of the spine (L1-L4) were observed.

A group by month interaction was observed for fat, lean and total body mass (Table 8). Fat mass for the vibration group was significantly lower compared to control, towards the end of the vibration period (−776.06 g, *p* = 0.043). For lean body mass, a reduction at month-12 compared to baseline produced a difference of (−519.11 g, *p* = 0.009) between the vibration group compared to control. Total body mass decreased from baseline to month-12 with group differences of (−1,285.72 g, *p* = 0.003) compared to control. Although not statistically significant, differences from month-18 to baseline demonstrated that these effects were not long lasting.

### Tibia high-resolution peripheral quantitative computed tomography

All tibia HR-pQCT parameters are presented in (Table 5). No significant improvement in total BMD compared to control was observed throughout the vibration phase (*p* = 1.00). No apparent differences could be observed for trabecular volume resulting from vibration exposure. In contrast, BV/TV exhibited a group by month interaction in the vibration group when comparing baseline to month-12 (*p* = 0.023). However, despite this significant difference between groups, the overall change reflected in BV/TV was negligible (0.0009%, *p* = 0.050). Trabecular inhomogeneity remained unchanged. No improvement in cortical area or thickness was observed. Apart from a gradual decrease over time no differences in cortical vBMD and perimeter were noted between groups. Finally, cortical porosity continued to increase over time in both the control and training group. Total muscle volume continued to decrease over time and MV/TV showed no overall differences between groups.

**TABLE 5:** Summary of all HR-pQCT parameters at the distal tibia.

	Control					Vibration						
	Contrast	% change	*p*-value	95% lower bound	95% upper bound	Contrast	% change	*p*-value	95% lower bound	95% upper bound	*p*-value group*month	*p*-value month
Total BMD (mgHA/cm^3^)											*p* = 0.0063	*p* = 0.149
Month 6 vs. Baseline	0.108	0.04	1.000	−1.107	1.323	−1.009	−0.40	0.154	−2.201	0.184		
Month 12 vs. Baseline	−1.841	−0.71	0.023	−3.517	−0.164	−1.346	−0.53	0.014	−2.509	−0.183		
Month 18 vs. Baseline	−2.186	−0.85	0.007	−3.970	−0.402	−3.187	−1.26	0.000	−4.565	−1.809		
Trabecular vBMD (mgHA/cm^3^)											*p* = 0.0159	*p* = 0.076
Month 6 vs. Baseline	0.257	0.17	1.000	−0.508	1.021	0.082	0.05	1.000	−0.675	0.839		
Month 12 vs. Baseline	−0.828	−0.54	0.165	−1.819	0.163	0.290	0.19	1.000	−0.456	1.036		
Month 18 vs. Baseline	−0.769	−0.50	0.576	−1.989	0.450	−0.198	−0.13	1.000	−1.116	0.721		
BV/TV (%)											*p* = 0.0231	*p* = 0.049
Month 6 vs. Baseline	0.000	0.17	1.000	0.000	0.001	0.000	0.10	1.000	−0.001	0.001		
Month 12 vs. Baseline	−0.001	−0.52	0.213	−0.002	0.000	0.000	0.23	1.000	0.000	0.001		
Month 18 vs. Baseline	−0.001	−0.52	0.506	−0.002	0.000	0.000	−0.11	1.000	−0.001	0.001		
Trabecular inhomogeneity (Tb.1/N.SD; mm)											*p* = 0.3615	*p* = 0.149
Month 6 vs. Baseline	0.002	0.57	1.000	−0.006	0.009	0.005	1.47	1.000	−0.009	0.019		
Month 12 vs. Baseline	0.003	1.17	1.000	−0.011	0.018	0.017	4.91	0.159	−0.003	0.038		
Month 18 vs. Baseline	0.005	1.69	1.000	−0.007	0.017	0.010	2.90	0.962	−0.009	0.029		
Cortical area (mm^2^)											*p* = 0.1317	*p* = 0.822
Month 6 vs. Baseline	0.359	0.37	1.000	−1.247	1.966	0.127	0.14	1.000	−0.857	1.110		
Month 12 vs. Baseline	−0.306	−0.32	1.000	−1.837	1.226	0.267	0.29	1.000	−0.798	1.332		
Month 18 vs. Baseline	0.470	0.49	1.000	−0.932	1.873	−0.208	−0.22	1.000	−1.326	0.911		
Cortical vBMD (mgHA/cm^3^)											*p* = 0.9484	*p* = 0.049
Month 6 vs. Baseline	−3.907	−0.49	0.034	−7.637	−0.176	−6.048	−0.75	0.000	−9.305	−2.790		
Month 12 vs. Baseline	−6.078	0.76	0.002	−10.503	−1.654	−9.481	−1.18	0.000	−13.111	−5.851		
Month 18 vs. Baseline	−9.814	−1.23	0.000	−14.344	−5.284	−13.523	−1.68	0.000	−17.747	−9.299		
Cortical perimeter (mm)											*p* = 0.3602	*p* = 0.065
Month 6 vs. Baseline	−0.155	−0.16	0.216	−0.349	0.040	−0.147	−0.15	0.280	−0.341	0.048		
Month 12 vs. Baseline	0.790	0.79	1.000	−1.792	3.371	−0.413	−0.41	0.001	−0.691	−0.136		
Month 18 vs. Baseline	−0.258	−0.26	0.082	−0.534	0.018	−0.408	−0.41	0.002	−0.709	−0.107		
Cortical thickness (mm)											*p* = 0.3772	*p* = 0.972
Month 6 vs. Baseline	−0.001	−0.05	1.000	−0.014	0.013	−0.001	−0.09	1.000	−0.012	0.011		
Month 12 vs. Baseline	−0.001	−0.13	1.000	−0.015	0.012	0.002	0.22	1.000	−0.009	0.014		
Month 18 vs. Baseline	0.000	0.01	1.000	−0.012	0.012	0.000	−0.03	1.000	−0.013	0.013		
Cortical porosity (%)											*p* = 0.6070	*p* = 0.559
Month 6 vs. Baseline	0.001	1.52	1.000	−0.002	0.005	0.002	2.81	0.094	0.000	0.004		
Month 12 vs. Baseline	0.003	3.79	0.147	−0.001	0.007	0.005	6.50	0.000	0.002	0.007		
Month 18 vs. Baseline	0.005	6.23	0.003	0.001	0.009	0.006	7.82	0.000	0.003	0.008		
Ultimate load (N)											*p* = 0.8138	*p* = 0.145
Month 6 vs. Baseline	30.062	−0.37	1.000	−65.523	125.647	70.369	−0.88	0.299	−24.280	165.017		
Month 12 vs. Baseline	74.798	−0.93	0.484	−38.158	187.754	86.170	−1.07	0.312	−30.849	203.190		
Month 18 vs. Baseline	61.717	−0.77	0.883	−50.621	174.056	131.971	−1.65	0.019	14.108	249.835		
Trabecular load vs. whole bone load (distal)											*p* = 0.4204	*p* = 0.016
Month 6 vs. Baseline	−0.006	−1.13	0.278	−0.014	0.002	−0.005	−0.90	0.566	−0.012	0.003		
Month 12 vs. Baseline	−0.002	−0.46	1.000	−0.011	0.006	−0.011	−2.07	1.000	−0.033	0.011		
Month 18 vs. Baseline	−0.006	−1.18	0.363	−0.015	0.002	−0.001	−0.27	1.000	−0.010	0.007		
Trabecular load vs. whole bone load (proximal)											*p* = 0.4699	*p* = 0.185
Month 6 vs. Baseline	−0.004	−1.30	0.872	−0.012	0.003	−0.002	−0.66	1.000	−0.009	0.005		
Month 12 vs. Baseline	0.000	−0.03	1.000	−0.008	0.008	−0.009	−2.63	0.925	−0.025	0.007		
Month 18 vs. Baseline	−0.003	−0.85	1.000	−0.012	0.007	−0.001	−0.34	1.000	−0.009	0.007		
Trabecular Von Mises stress (MPa)											*p* = 0.8662	*p* = 0.156
Month 6 vs. Baseline	−0.473	−0.90	1.000	−1.566	0.619	−0.573	−1.06	1.000	−1.762	0.616		
Month 12 vs. Baseline	−0.379	−0.72	1.000	−1.595	0.837	−1.270	−2.35	0.516	−3.222	0.682		
Month 18 vc Baseline	−0.487	−0.92	1.000	−1.793	0.819	−1.022	−1.89	0.349	−2.445	0.401		
Cortical Von Mises stress (MPa)											*p* = 0.0927	*p* = 0.477
Month 6 vs. Baseline	−0.135	−0.16	1.000	−0.431	0.162	−0.179	−0.21	0.334	−0.425	0.068		
Month 12 vs. Baseline	−0.232	−0.27	0.194	−0.518	0.054	0.011	0.01	1.000	−0.295	0.318		
Month 18 vs. Baseline	−0.146	−0.17	1.000	4.446	0.155	−0.226	−0.26	0.588	−0.586	0.134		
Total Muscle Volume (mm^3^)											*p* = 0.5400	*p* = 0.008
Month 6 vs. Baseline	37.341	0.19	1.000	454252	528.935	−115.230	−0.61	1.000	450.794	220.333		
Month 12 vs. Baseline	166.399	0.85	1.000	−356.981	689.779	−166.633	−0.89	1.000	−536.316	203.050		
Month 18 vs. Baseline	−117.924	−0.60	1.000	−471.104	235.255	−256.935	−1.37	0.224	−582.550	68.679		
MV/TV											*p* = 0.1708	*p* = 0.914
Month 6 vs. Baseline	−0.002	−0.41	1.000	−0.011	0.007	0.000	−0.04	1.000	−0.007	0.006		
Month 12 vs. Baseline	−0.009	−1.62	0.062	−0.018	0.000	0.001	0.13	1.000	−0.007	0.008		
Month 18 vs. Baseline	−0.006	−1.11	0.776	−0.017	0.005	−0.003	−0.51	1.000	−0.011	0.005		

### Radius high-resolution peripheral quantitative computed tomography

All radius HR-pQCT parameters are presented in (Table 6). No improvement in total bone mineral density was evident. Trabecular volume, BV/TV as well as trabecular inhomogeneity remained unaltered. No significant interactions were observed for any cortical parameters. For cortical area, thickness, perimeter and cortical vBMD no improvement resulting from vibration could be detected. Finally, both groups experienced an increase in cortical porosity levels over time.

**TABLE 6:** Summary of all HR-pQCT parameters at the distal radius.

	Control					Vibration						
	Contrast	% change	*p*-value	95% lower bound	95% upper bound	Contrast	% change	*p*-value	95% lower bound	95% upper bound	*p*-value group*month	*p*-value month
Total BMD (mgHA/cm^3^)											*p* = 0.4353	*p* = 0.092
Month 6 vs. Baseline	0.51	0.17	1.00	−1.87	2.89	−0.95	−0.32	1.00	−3.25	1.36		
Month 12 vs. Baseline	−1.93	−0.65	0.25	−4.43	0.58	−1.73	−0.59	0.58	−4.47	1.01		
Month 18 vs. Baseline	−3.71	−1.25	0.08	−7.66	0.25	−4.09	−1.39	0.00	−6.92	−1.26		
Trabecular vBMD (mgHA/cm^3^)											*p* = 0.5609	*p* = 0.010
Month 6 vs. Baseline	0.04	0.03	1.00	−0.98	1.06	0.10	0.07	1.00	−0.95	1.15		
Month 12 vs. Baseline	−0.75	−0.50	0.48	−1.89	0.38	0.25	0.17	1.00	−1.00	1.50		
Month 18 vs. Baseline	−2.28	−1.52	0.12	−4.85	0.29	−0.78	−0.54	0.83	−2.16	0.61		
BV/TV (%)											*p* = 0.0112	*p* = 0.011
Month 6 vs. Baseline	0.00	0.05	1.00	0.00	0.00	0.00	0.09	1.00	0.00	0.00		
Month 12 vs. Baseline	0.00	−0.54	0.39	0.00	0.00	0.00	0.22	1.00	0.00	0.00		
Month 18 vs. Baseline	0.00	−1.52	0.11	0.00	0.00	0.00	−0.53	0.88	0.00	0.00		
Trabecular inhomogeneity (Tb.1/N.SD; mm)											*p* = 0.1465	*p* = 0.037
Month 6 vs. Baseline	0.01	2.45	0.27	0.00	0.02	0.01	4.13	0.02	0.00	0.03		
Month 12 vs. Baseline	0.00	1.49	1.00	−0.01	0.02	0.01	3.30	1.00	−0.01	0.03		
Month 18 vs. Baseline	0.01	2.52	0.27	0.00	0.02	0.01	4.13	0.26	0.00	0.03		
Cortical area (mm^2^)											*p* = 0.3697	*p* = 0.260
Month 6 vs. Baseline	0.37	0.75	0.88	−0.30	1.04	−0.19	−0.39	1.00	−0.73	0.36		
Month 12 vs. Baseline	−0.02	−0.03	1.00	−0.80	0.77	−0.14	−0.28	1.00	−0.69	0.42		
Month 18 vs. Baseline	1.51	3.10	1.00	−2.81	5.84	−0.55	−1.14	0.18	−1.23	0.12		
Cortical vBMD (mgHA/cm^3^)											*p* = 0.4968	*p* = 0.582
Month 6 vs. Baseline	−0.92	−0.11	1.00	−5.36	3.51	−3.55	−0.41	0.22	−8.05	0.94		
Month 12 vs. Baseline	−3.86	−0.46	0.40	−9.43	1.70	−6.90	−0.80	0.00	−11.65	−2.15		
Month 18 vs. Baseline	−11.69	−1.38	0.52	−29.67	6.28	−7.65	−0.89	0.00	−12.45	−2.85		
Cortical perimeter (mm)											*p* = 0.1963	*p* = 0.056
Month 6 vs. Baseline	−1.37	−1.99	1.00	−4.18	1.45	−0.08	−0.11	1.00	−0.31	0.16		
Month 12 vs. Baseline	−1.49	−2.17	0.97	−4.30	1.32	−0.23	−0.35	0.04	−0.45	−0.01		
Month 18 vs. Baseline	−1.54	−2.24	0.89	−4.35	1.27	−0.36	−0.55	0.00	−0.61	−0.12		
Cortical thickness (mm)											*p* = 0.1879	*p* = 0.616
Month 6 vs. Baseline	0.00	0.55	1.00	−0.01	0.02	−0.01	−0.71	0.30	−0.01	0.00		
Month 12 vs. Baseline	0.00	−0.10	1.00	−0.01	0.01	0.00	−0.30	1.00	−0.01	0.01		
Month 18 vs. Baseline	0.01	1.49	1.00	−0.03	0.06	−0.01	−0.71	0.86	−0.02	0.00		
Cortical porosity (%)											*p* = 0.7435	*p* = 0.059
Month 6 vs. Baseline	0.00	7.22	0.35	0.00	0.00	0.00	8.05	0.05	0.00	0.00		
Month 12 vs. Baseline	0.00	8.08	0.21	0.00	0.00	0.00	8.74	0.06	0.00	0.00		
Month 18 vs. Baseline	0.01	56.38	0.98	−0.01	0.04	0.00	14.13	0.00	0.00	0.00		
Ultimate load (N)											*p* = 0.3537	*p* = 0.005
Month 6 vs. Baseline	18.16	−0.64	1.00	−55.29	91.61	52.41	−1.81	0.94	−45.08	149.90		
Month 12 vs. Baseline	80.07	−2.80	0.00	16.85	143.28	64.08	−2.21	0.01	11.54	116.62		
Month 18 vs. Baseline	39.49	−1.38	0.93	−33.88	112.87	86.96	−3.00	0.00	25.80	148.13		
Trabecular load vs. whole bone load (distal)											*p* = 0.4963	*p*<0.001
Month 6 vs. Baseline	0.00	−0.39	1.00	−0.02	0.01	0.00	0.81	1.00	−0.01	0.02		
Month 12 vs. Baseline	−0.01	−2.84	0.16	−0.03	0.00	−0.01	−2.49	0.39	−0.03	0.00		
Month 18 vs. Baseline	0.01	1.41	1.00	−0.01	0.02	0.00	−0.10	1.00	−0.02	0.02		
Trabecular load vs. whole bone load (proximal)											*p* = 0.3569	*p* = 0.008
Month 6 vs. Baseline	0.00	−3.03	0.66	−0.01	0.00	0.00	0.69	1.00	−0.01	0.01		
Month 12 vs. Baseline	−0.01	−3.52	0.49	−0.01	0.00	0.00	−2.17	1.00	−0.01	0.00		
Month 18 vs. Baseline	0.00	0.40	1.00	−0.01	0.01	0.00	0.43	1.00	−0.01	0.01		
Trabecular Von Mises stress (MPa)											*p* = 0.8640	*p* = 0.009
Month 6 vs. Baseline	−0.14	−0.34	1.00	−1.66	1.37	−0.27	−0.62	1.00	−1.99	1.45		
Month 12 vs. Baseline	−1.44	−3.38	0.11	−3.05	0.17	−1.08	−2.48	0.33	−2.56	0.40		
Month 18 vs. Baseline	0.02	0.06	1.00	−1.64	1.68	−0.62	−1.43	1.00	−2.24	0.99		
Cortical Von Mises stress (MPa)											*p* = 0.4670	*p* = 0.175
Month 6 vs. Baseline	−0.34	−0.43	1.00	−1.25	0.57	−1.15	−1.44	1.00	−3.38	1.08		
Month 12 vs. Baseline	−0.70	−0.89	0.27	−1.63	0.22	−0.41	−0.51	0.94	−1.16	0.35		
Month 18 vs. Baseline	−0.92	−1.16	0.40	−2.23	0.40	−0.71	−0.88	0.06	−1.43	0.02		

### Finite element analysis

All finite element parameters for the radius and tibia are presented in (Tables 5, 6). WBV failed to improve finite element parameters in the vibration group. Failure load continued to worsen over time in both the tibia and radius. The ratio of the load supported by the trabecular bone and the load by the whole bone at the distal tibial site continued to decrease (*p* = 0.016) throughout. In contrast, these changes were not significantly different at the proximal end (*p* = 0.185). For the radius, both the distal and proximal sites showed reductions over time with values of (*p* < 0.001) and (*p* = 0.008), respectively. Cortical Von Mises stress for the tibia and radius moderately decreased over time, however these changes were not statistically significant. Trabecular Von Mises stress on the other hand demonstrated no significant changes over time for the tibia, however, significant decreases in stress loads over time were seen for the radius (*p* = 0.009).

### Serum analysis

**FIGURE 3:**
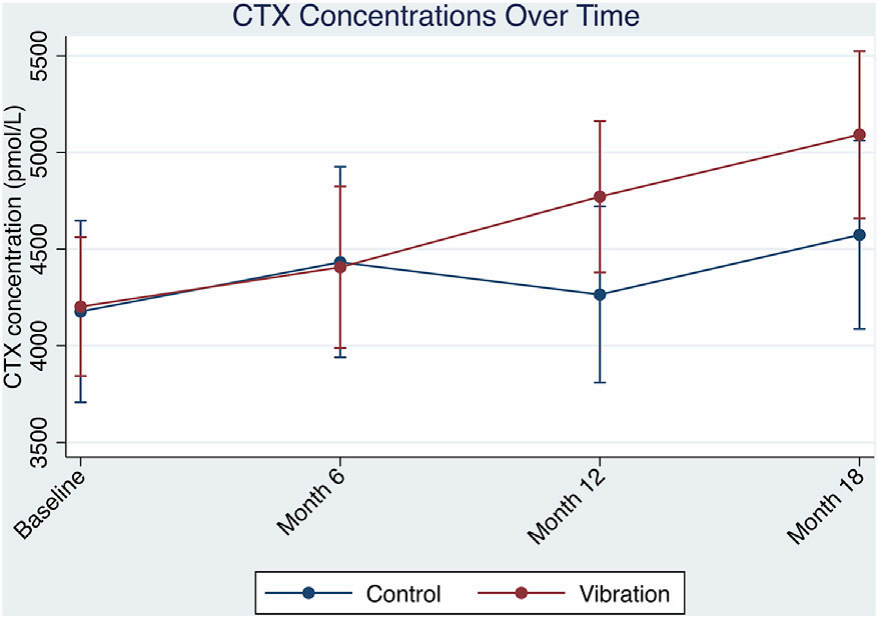
Group by month interaction for serum CTX comparing control (*n* = 98) and vibration (*n* = 99) groups. Values depicted are estimated marginal means derived from the linear mixed model plotted with 95% CI’s. Vibration period (Baseline to Month-12) in the vibration group and regular visits for the control group (Baseline to Month-12). Follow-up visits for both groups (Month-12–Month-18).

All serum markers are presented in (Table 7). Interaction effects between month and group were observed for serum CTX (*p* = 0.015) (Table 8; Figure 3). Although both groups experienced an increase in CTX concentrations, a more marginal elevation was noted in the vibration group. Compared to baseline, the vibration group at month 12 produced greater differences in CTX concentrations compared to control (480.80 pmol/L; *p* = 0.039). However, no differences between groups were observed when only comparing groups at month-12 (506.35 pmol/L; *p* = 0.415) and month-18 (518.33 pmol/L; *p* = 0.480). No significant differences in sclerostin concentration could be observed. Apart from significant increases over time (*p* < 0.001), no other differences were observed for BAP. Where P1NP was concerned, no significant alterations were observed.

**TABLE 7:** Summary of all serum parameters.

	Control					Vibration						
	Contrast	% change	*p*-value	95% lower bound	95% upper bound	Contrast	% change	*p*-value	95% lower bound	95% upper bound	*p*-value group*month	*p*-value month
PINP (ug/L)											*p* = 0.3254	*p* = 0.509
Month 6 vs. Baseline	0.23	0.41	1.00	−2.34	2.79	−0.76	−1.36	1.00	−3.80	2.27		
Month 12 vs. Baseline	−0.43	−0.76	1.00	−4.07	3.22	1.05	1.88	1.00	−2.64	4.74		
Month 18 vs. Baseline	−0.61	−1.10	1.00	−5.11	3.88	−2.03	−3.61	1.00	−6.27	2.21		
CTX (pmol/L)											*p* = 0.0155	*p* < 0.001
Month 6 vs. Baseline	223.34	5.39	0.39	−95.26	541.94	169.85	3.92	1.00	−161.59	501.28		
Month 12 vs. Baseline	25.42	0.61	1.00	−326.68	377.52	506.29	11.68	0.00	136.70	875.88		
Month 18 vs. Baseline	304.45	7.35	021	−77.31	686.22	797.43	18.40	0.00	361.07	1233.79		
BAP (ug/L)											*p* = 0.3818	*p* < 0.001
Month 6 vs. Baseline	1.29	10.57	0.00	0.70	1.89	1.21	10.31	0.00	0.53	1.88		
Month 12 vs. Baseline	2.37	19.33	0.00	1.56	3.17	2.51	21.41	0.00	1.71	3.31		
Month 18 vs. Baseline	3.48	28.45	0.00	2.47	4.50	2.87	24.47	0.00	1.95	3.78		
Sclerostin (ng/mL)											*p* = 0.0881	*p* = 0.772
Month 6 vs. Baseline	0.01	1.96	0.89	−0.01	0.04	−0.01	−1.62	1.00	−0.03	0.02		
Month 12 vs. Baseline	0.00	−0.36	1.00	−0.03	0.02	0.00	0.65	1.00	−0.02	0.03		
Month 18 vs. Baseline	0.01	1.11	1.00	−0.02	0.03	0.00	0.73	1.00	−0.02	0.03		

**TABLE 8:** Interaction effects table.

	Control		Vibration						
	Contrast	% change	Contrast	% change	Difference compared to vibration group	*p*-value	95% lower bound	95% upper bound	*p*-value group*month
DXA Fat mass (g)									*p* = 0.0107
Month 6 vs. Baseline	268.74	1.01	−47.37	−0.19	−316.11	0.573	−894.894	262.665	
Month 12 vs. Baseline	752.91	2.82	−23.16	−0.09	−776.06	0.043	−1535.777	−16.35	
Month 18 vs. Baseline	326.41	1.22	267.62	1.07	−58.79	1.000	−846.76	729.182	
Lean mass (g)									*p* = 0.0268
Month 6 vs. Baseline	501.40	1.33	146.61	0.40	−354.79	0.132	−776.399	66.814	
Month 12 vs. Baseline	837.14	2.21	318.02	0.86	−519.12	0.009	−936.298	−101.941	
Month 18 vs. Baseline	697.33	1.84	415.26	1.13	−282.07	0.390	−728.222	164.085	
Body mass (g)									*p* = 0.0015
Month 6 vs. Baseline	766.60	1.15	98.48	0.15	−668.11	0.121	−1448.131	111.907	
Month 12 vs. Baseline	1577.93	2.37	292.20	0.46	−1285.72	0.003	−2223.718	−347.732	
Month 18 vs. Baseline	1005.83	1.51	674.80	1.05	−331.03	1.000	−1270.346	608.277	
Tibia HR-pQCT Total BMD (mgHA/cm^3^)									*p* = 0.0063
Month 6 vs. Baseline	0.64	0.25	−0.46	−0.18	−1.09151	0.272	−2.637	0.454	
Month 12 vs. Baseline	−0.81	−0.31	−0.32	−0.13	0.49332	1.000	−1353	2.34	
Month 18 vs. Baseline	−0.65	−0.25	−1.67	−0.66	−1.02255	0.686	−3.056	1.011	
Trabecular vBMD (mgHA/cm^3^)									*p* = 0.0159
Month 6 vs. Baseline	−0.02	−0.01	−0.21	−0.13	−0.18782	1.000	−1.167	0.791	
Month 12 vs. Baseline	−1.37	−0.89	−0.25	−0.16	1.11947	0.052	−0.008	2.247	
Month 18 vs. Baseline	−1.57	−1.02	−0.99	−0.64	0.58315	0.941	−0.803	1.969	
BVTV (%)									*p* = 0.0231
Month 6 vs. Baseline	0.00	−0.02	−0.00011	−0.09	−0.00009	1.000	−0.001	0.001	
Month 12 vs. Baseline	0.00	−0.88	−0.00017	−0.13	0.00096	0.050	0.000	0.002	
Month 18 vs. Baseline	0.00	−1.06	−0.00082	−0.64	0.00054	0.825	−0.001	0.002	
Serum CTX (pmol/L)									*p* = 0.0155
Month 6 vs. Baseline	255.47	6.12	203.73	4.85	−51.74	1.000	−468.701	365.225	
Month 12 vs. Baseline	87.79	2.10	568.60	13.53	480.8	0.039	17.735	943.873	
Month 18 vs. Baseline	397.14	9.51	889.92	21.18	492.78	0.075	−33.247	1018.812	

## Discussion

This non-randomised clinical trial matched participants according to their FRAX scores for major osteoporotic fracture (MOF) 10 years-absolute risk ranging from (3%–35%) to ensure group comparability investigated the effects of WBV on 197 postmenopausal women over 12 months, with an additional 6-month follow-up to observe post-vibratory outcomes. Treatment responses were measured using DXA, HR-pQCT, and serum analysis. Fat, lean body, and total body mass assessed by DXA very marginally decreased in the vibration group during the vibration phase. No significant differences at the femoral neck or for all other DXA and serum parameters were observed except for an 11% increase in CTX levels in the vibration group, a non-clinically relevant variation which remained present at month-18. Furthermore, no demonstrable benefits from vibration were detected for all bone geometry and microarchitectural parameters measured by HR-pQCT. Finite element analysis demonstrated a continuous decrease in failure load, and disproportionate stress loads were observed for the cortical and trabecular compartments in both groups. Finally, no changes in muscle parameters evaluated by HR-pQCT at the distal tibia were detected.

The primary objective was to assess the effect of WBV on the femoral neck BMD, given that this parameter is a strong predictor of hip and spine fractures in this type of population (Crandall et al., 2021). Some studies have found neutral effects of WBV at the hip and femoral neck (Lau et al., 2011; Ma et al., 2016; Oliveira et al., 2016; Jepsen et al., 2017; Luo et al., 2017) while one demonstrated positive effects (Sitjà-Rabert et al., 2012). Although the objective of previous studies has been on frequency variation and exposure length, none have incorporated combined amplitude in the experimental protocol. Therefore, unique to this study was the incorporation of high and low-amplitude stimuli with an incremental increase throughout the vibration protocol. By further tailoring the signal characteristics that have generally been sufficient to stimulate bone formation (Santos et al., 2017), this protocol was expected to better replicate the role of mechanical stimulation, in particular the ground reaction forces produced during regular jumping and physical activity and to demonstrate its tolerance in this population. However, despite this combination of amplitude and frequency, no discernible benefits were observed at the femoral neck, spine, or HR-pQCT parameters. The continued reduction in failure load with unequal load distributions between cortical and trabecular compartments is consistent with elevated fracture risk (Boutroy et al., 2008) in this population, not significantly negated by vibration therapy.

The results from this study are difficult to compare, given the unique signal characteristics. However, considering studies 12 months in duration, Slatkovska et al. (2011) share several similarities (population size, age range and 20-min training sessions with predominantly European participants) with the current study. Although Slatkovska et al. (2011) mainly focused on frequency variation (90 Hz and 30 Hz), the frequency range in the present study (30 Hz–50 Hz) fell between the two training groups. This is a particular strength as it helps to further expand upon the range of frequencies previously tested while incorporating the application of combined amplitude. However, both Slatkovska et al. (2011) and the present study failed to see any benefits on bone after 12 months of WBV exposure. Another one-year longitudinal study (Liphardt et al., 2015), which incorporated a follow-up period to observe sustained osteogenic benefits of WBV, also failed to detect changes in the bone and observed no improvement in load distribution between groups, which indicates no improvement in fracture risk. Though these studies employed different platforms and signal attributes, the consistency in HR-pQCT results of bone geometry and microarchitecture remains a crucial finding.

The current results can be further contrasted with other studies using the PowerPlate device. Sen et al. (2020) and Verschueren et al. (2004) utilised high-frequency stimuli for 6 months to measure BMD changes at one or more sites (hip, femoral neck, and lumbar region) along with serum bone turnover markers (CTX and osteocalcin). In both studies, the vibration groups demonstrated an improvement in BMD; however, bone turnover marker results were inconsistent. In the present study, no significant benefits were observed at the crucial DXA sites. However, from baseline to the end of the training period, CTX was elevated in both groups with marginally higher concentrations in the vibration group. Nevertheless, when comparing months 12 and 18 alone these differences were not statistically different.

Several explanations could account for the marginal increase in CTX concentrations. Firstly, the two previous studies (Verschueren et al., 2004; Sen et al., 2020) were of much shorter duration, hence CTX would not have changed as it did in this study. Secondly, when taken together, the increased BAP activity with the rising CTX concentrations is indicative of the stimulated bone remodeling process in postmenopausal women (Eastell et al., 2016). Finally, the marginal elevation in CTX concentrations in the vibration group could also be linked with the small reduction in body mass resulting from WBV exposure. The impact of weight loss attributed to dieting and/or exercise on bone turnover markers (CTX) has been previously reported (Eastell and Szulc, 2017).

Sen et al. (2020) and Verschueren et al. (2004) examined the response of WBV on osteocalcin, as a bone formation marker. No group differences were reported by Verschueren et al. (2004) while a decrease was reported by Sen et al. (2020). In the present study, the effects on bone formation were studied using P1NP which was not altered due to vibration training. Elevation in P1NP was observed by Corrie et al. (2015) in a study using a PowerPlate device. However, the age of the participants was considerably higher (79–82 years) than the current study, the training duration was only twelve weeks and focused on both men and women in a small population. It is also important to note that no BMD measurements were taken during this study.

WBV response on bone turnover markers has not been extensively studied, however, the few that have investigated its effects (Russo et al., 2003; Verschueren et al., 2004; Corrie et al., 2015; Kiel et al., 2015) focused mainly on markers of formation and resorption. Even fewer have explored the role of sclerostin in the context of WBV. Sclerostin has emerged over the years as a marker essential to skeletal physiology and homeostasis and is found to be mostly expressed by osteocytes (Poole et al., 2005). It interacts with the Wnt signalling pathway (Baron and Kneissel, 2013) leading to the formation or resorption of bone. Moreover, sclerostin concentrations have been shown to increase or decrease based on the response to mechanical stimuli (Robling et al., 2008) and are also known to increase with age (Amrein et al., 2012). The goal of measuring sclerostin was to evaluate if the signal characteristics in this study could be detected. Since no differences between groups were observed, this further highlights that the effects of combined amplitude stimuli were undetected. However, it does pose the question of whether combined amplitude exposure of longer duration could produce significant group differences.

Previous research has also explored age-related responses to WBV (Slatkovska et al., 2011; Marín-Cascales et al., 2018); however, the relevance within the context of high-frequency and combined amplitude training has not been tested. Moreover, since fracture risk increases substantially beyond age 64 (Arlot et al., 1997; Emaus et al., 2006), this breakpoint provides an opportunity to ascertain dose and bone response associated with age, resulting from WBV exposure. These age-related differences were explored using retrospective statistical analysis by incorporating age groups (≤ 64 and > 64) into the fixed effects of the aforementioned statistical model to further examine whether any effects of WBV could be detected (Supplementary Material S8–S11). This was done to elucidate any preventative effects given that WBV is relied upon more as a preventive treatment modality. Despite such attempts, no significant differences between age groups were noted. Both bone and serum parameters demonstrated no significant improvement, and more importantly, parameters including P1NP, which is a marker sensitive to bone formation (Gillett et al., 2021), exhibited no significant improvement resulting from vibration therapy. As a result, this further substantiates that WBV has neither osteogenic benefits nor negates fracture risk in this type of physically inactive population regardless of the signal characteristics.

The minimal reductions in fat, lean body and total body mass observed towards the end of the vibration protocol could be linked with a few different possibilities. Firstly, the vibration group was aware that they were undergoing treatment, and the possible impact of this awareness cannot be discounted as an influencing factor. As a result, participants could have been influenced in their dietary habits. Nevertheless, aside from this possibility, the results indicate an interesting effect between groups in that the control group saw more significant weight increases over time than the vibration group. The present study is not the only one to report this effect since the impact of vibration on weight has previously been reported by many (Cristi-Montero, 2013). Several hypotheses exist and involve the role of the sympathetic nervous system (Alavinia et al., 2021), osteocalcin and sclerostin (Wang et al., 2021). A recent pilot study also suggested the role of SMP30 on fat mass, given its role in lipid regulation (Pérez-Gómez et al., 2020). Further studies are needed to fully understand the mechanism as it remains largely unknown and poorly understood.

## Limitations

To the best of our knowledge, this study was the first to comprehensively investigate the effects of WBV by including an additional 6-month follow-up period to examine bone geometry and microarchitecture, muscle (HR-pQCT distal tibia), serum and DXA with a particular focus on the femoral neck, using high-frequency and combined amplitude training. Despite this, there are several limitations:

1) This study investigated the effects of WBV in a group of Caucasian European postmenopausal women; however, future research will have to explore the effect of menopause on WBV using broader clinical criteria by accounting for the age, stage of menopause, level of exercise and diverse ethnicities.
2) This study was primarily designed to observe the overall effects of WBV on a large physically inactive group and was not specifically tailored to extract age-related responses. However, given the large population, age range, and adherence to the study protocol, a retrospective statistical analysis, showed that 12 months of WBV treatment did not produce any preventative effects specific to the bone.
3) Finally, the exercise profile of all participants at recruitment relied on several questionnaires to account for their physical activity levels. While this provided a fair estimate of both their general and physical activity levels at recruitment, future studies will need to select parameters involving a combination of physiological and questionnaire-based measures to attain a more accurate and comprehensive assessment.
4) In future studies, closer nutritional monitoring between groups along with the use of Magnetic Resonance Imagining (MRI) could help to assess the effects of WBV on weight.

## Conclusion

In conclusion, this 18-month non-randomised clinical trial matched participants according to their (MOF) FRAX scores for major osteoporotic fracture and used WBV for 12 months with combined amplitude and high-frequency vibration to improve bone geometry and bone microarchitecture. This study failed to significantly detect an improvement in bone outcomes of physically inactive osteopenic postmenopausal women. Although the 12-month training protocol was well tolerated, DXA BMD measurements of the lumbar spine, femoral neck and hip were not significantly improved compared to control. Moreover HR-pQCT analysis of cortical and trabecular compartments, including finite element analysis did not demonstrate significant improvement resulting from WBV training. Serum markers (P1NP, BAP and sclerostin) also showed no response to mechanical stimuli. A marginal increase in CTX concentrations was observed; however, there was no indication that this could have resulted from vibration exposure. Nevertheless, future studies could expand upon these results by accounting for stratification in age, and stage of menopause to draw further conclusions.

## Data Availability

The original contributions presented in the study are included in the article/Supplementary Material, further inquiries can be directed to the corresponding author.
