# Supplementary material for: The effects of combined amplitude and high-frequency vibration on physically inactive osteopenic postmenopausal women
Source: Front Physiol. 2022 Sep 7;13:952140. doi: 10.3389/fphys.2022.952140 (PMC9491321; doi:10.3389/fphys.2022.952140)
Supplement: Supplementary file 1 [file Table1.DOCX]

**SUPPLEMENTAL DATA 1:** QUANTAP questionnaire

**General information**

Date: ...................................

Full name: ............................... ............................

Month and year of birth: ................................................

Weight: ....................kg Height: ...................m

**EDUCATIONAL ACTIVITIES (SCHOOLS)**

Entry to ^1st^ degree (year or age): ....................................

Entry into the 2nd degree (year or age): ....................................

Exit (year or age): ..............................

**PRE-PROFESSIONAL ACTIVITIES (Before your final professional activity)**

Activity: ...........................................................................

Year or age at start: ............... Year or age at end: ................

Type of Physical Activity: 1: light 2: Moderate 3: Intense

Number of hours per week:

1: 0 to 1h

2: Moderate 2: 1 to 3 hours

3: Hard 3: 3 to 5h

4: very hard 4: 5 to 10 hours

5: Maximal 5: + 10h

Activity: ...........................................................................

Starting year or age: ................. End year or age: ...............

Type of Physical Activity: 1: light 2: Moderate 3: Intense

Number of hours per week:

1: 0 to 1h

2: Moderate 2: 1 to 3 hours

3: Hard 3: 3 to 5h

4: very hard 4: 5 to 10 hours

5: Maximal 5: + 10h

Activity: ...........................................................................

Starting year or age: ................. End year or age: ..................

Type of Physical Activity: 1: light 2: Moderate 3: Intense

Number of hours per week:

1: 0 to 1h

2: Moderate 2: 1 to 3 hours

3: Hard 3: 3 to 5h

4: very hard 4: 5 to 10 hours

5: Maximal 5: + 10h

**PROFESSIONAL ACTIVITY**

Are you retired (circle the correct answer): YES / NO

If YES, since what year or age: ......................

Title of your professional activity: ................................................

Number of hours/week: ........................

Year or age at start:........................ Year or age at end:...............

Working posture: Resting

Handling of work tools:

None

Sitting light

Sitting as well as standing average

Stand up painful

Weight of load carried: No load carried

Light load

Average load

Heavy load

Active travel : None

Very few

Often on flat ground sometimes uneven

Often with a difference in altitude

**SPORTS ACTIVITIES**

Activity: ...................................................

Supervised (circle the correct answer): YES/NO

Type (circle one): School/leisure/club

Year or start age: .............. Year or age end: ................

Characteristics of the sessions :

Duration: .......hr/s......min/s Number per month:......... Month/year:.........

Activity: ...................................................

Supervised (circle the correct answer): YES/NO

Type (circle one): School/leisure/club

Year or start age: .............. Year or age end: ................

Characteristics of the sessions :

Duration: .......hr/s......min/s Number per month:......... Month/year:.........

Activity: ...................................................

Supervised (circle the correct answer): YES/NO

Type (circle one): School/leisure/club

Year or start age: .............. Year or age end: ................

Characteristics of the sessions :

Duration: .......hr/s......min/s Number per month:......... Month/year:.........

Activity: ...................................................

Supervised (circle the correct answer): YES/NO

Type (circle one): School/leisure/club

Year or start age: .............. Year or age end: ................

Characteristics of the sessions :

Duration: .......hr/s......min/s Number per month:......... Month/year:.........

**DAILY ACTIVITY**

5: Very intense

4: Intense

3: Medium

2: Light

1: Very light

| Classes | Gardening | DIY | Household | Displacement | Music | Manual activity |
| --- | --- | --- | --- | --- | --- | --- |
| 1-10 years |  |  |  |  |  |  |
| 11-20 years |  |  |  |  |  |  |
| 21-30 years |  |  |  |  |  |  |
| 31-40 years |  |  |  |  |  |  |
| 41-50 years |  |  |  |  |  |  |
| 51-60 years |  |  |  |  |  |  |
| 61-70 years |  |  |  |  |  |  |
| 71-80 years |  |  |  |  |  |  |

**SUPPLEMENTAL DATA 2**: Vibration training protocol with combined high (0.6-0.8mm) and low (0.2-0.4mm) amplitude stimuli

| **WEEK(1)- ADAPTATION PERIOD- REST TIME BETWEEN EXERCISES- 60 SECONDS** | | | | | |
| --- | --- | --- | --- | --- | --- |
|  |  |  |  |  |  |
|  | **POSITION** | **TIME(mins,sec)** | **FREQUENCY (Hz)** | **AMPLITUDE** | **TOTAL PROTOCOL TIME** |
|  | Light squats | 1:30 | 30Hz | Low |  |
|  | Deep Squats | 2:30 | 35Hz | Low | 10:30 minutes |
|  | Balanced | 2:30 | 35Hz | Low |  |
|  | Stretching exercises | 1:60 | 50Hz | Low |  |
|  | Relaxation exercises | 1:60 | 50Hz | Low |  |
|  |  |  |  |  |  |
| **WEEK(2-4)- ADDITIONAL EXERCISES- REST TIME BETWEEN EXERCISES- 60 SECONDS** | | | | | |
|  |  |  |  |  |  |
|  | Light squats | 1:30 | 30Hz | Low |  |
|  | Deep Squats | 2:30 | 40Hz | Low |  |
|  | Forced squats | 1:30 | 40Hz | Low |  |
|  | Balanced | 1:30 | 40Hz | Low |  |
|  | Right leg exercises | 1:30 | 40Hz | Low | 15:30 minutes |
|  | Left leg exercises | 1:30 | 40Hz | Low |  |
|  | Core strength exercises | 1:30 | 40Hz | Low |  |
|  | Stretching exercises | 1:60 | 50Hz | Low |  |
|  | Relaxation exercises | 1:60 | 50Hz | Low |  |
|  |  |  |  |  |  |
| **WEEK(5-7)- FREQUENCY AUGMENTATION- REST TIME BETWEEN EXERCISES- 60 SECONDS** | | | | | |
|  |  |  |  |  |  |
|  | Light squats | 1:30 | 35Hz | Low |  |
|  | Deep Squats | 2:30 | 45Hz | Low |  |
|  | Forced squats | 2:30 | 45Hz | Low |  |
|  | Balanced | 1:30 | 45Hz | Low | 17:30 minutes |
|  | Right leg exercises | 1:30 | 45Hz | Low |  |
|  | Left leg exercices | 1:30 | 45Hz | Low |  |
|  | Abdominal exercises | 1:30 | 40Hz | Low |  |
|  | Stretching exercises | 2:60 | 50Hz | Low |  |
|  | Relaxation exercises | 1:60 | 50Hz | Low |  |
|  |  |  |  |  |  |
| **WEEKS(8-11)-EXERCISE AUGMENTATION- REST TIME BETWEEN EXERCISES- 60 SECONDS** | | | | | |
|  |  |  |  |  |  |
|  | Light squats | 1:45 | 35Hz | Low |  |
|  | Deep Squats | 2:45 | 45Hz | High |  |
|  | Forced squats | 1:45 | 45Hz | High |  |
|  | Balanced | 1:45 | 45Hz | High |  |
|  | Right leg exercises | 1:45 | 45Hz | Low | 20 minutes |
|  | Left leg exercises | 1:45 | 45Hz | Low |  |
|  | Chair squat | 1:45 | 45Hz | High |  |
|  | Abdominal exercises | 2:45 | 40hz | Low |  |
|  | Stretching exercises | 1:60 | 50Hz | Low |  |
|  | Relaxation exercises | 1:60 | 50Hz | Low |  |
|  |  |  |  |  |  |
| **WEEKS(12-14) - AUGMENTATION OF EXERCISE TIME- REST TIME BETWEEN EXERCISES- 60 SECONDS** | | | | | |
|  |  |  |  |  |  |
|  | Light squats | 1:45 | 35Hz | Low |  |
|  | Deep Squats | 2:60 | 45Hz | High |  |
|  | Forced squats | 1:60 | 45Hz | High |  |
|  | Balanced | 1:60 | 45Hz | High | 18:45 minutes |
|  | Core strength exercises | 1:60 | 45Hz | Low |  |
|  | Chair squat | 1:60 | 45Hz | Low |  |
|  | Abdominal exercises | 1:60 | 40Hz | Low |  |
|  | Stretching exercises | 1:60 | 50Hz | Low |  |
|  | Relaxation exercises | 1:60 | 50Hz | Low |  |
|  |  |  |  |  |  |
| **WEEKS(15-17)- AUGMENTATION OF REPETITIONS- REST TIME BETWEEN EXERCISES- 60 SECONDS** | | | | | |
|  |  |  |  |  |  |
|  | Light squats | 1:45 | 35Hz | Low |  |
|  | Deep Squats | 2:60 | 45Hz | High |  |
|  | Core strength exercises | 1:60 | 45Hz | High |  |
|  | Balanced | 1:60 | 45Hz | High |  |
|  | Right leg exercises | 1:60 | 45Hz | Low | 20:45 minutes |
|  | Left leg exercises | 1:60 | 45Hz | Low |  |
|  | Chair squat | 2:60 | 45Hz | Low |  |
|  | Abdominal exercises | 2:60 | 40hz | Low |  |
|  | Stretching exercises | 1:60 | 50Hz | Low |  |
|  | Relaxation exercises | 1:60 | 50Hz | Low |  |
|  |  |  |  |  |  |
| **WEEKS(18-21) - DYNAMIC EXERCISE PROTOCOL- REST TIME BETWEEN EXERCISES- 45 SECONDS** | | | | | |
|  |  |  |  |  |  |
|  | Light squats | 1:45 | 35Hz | Low |  |
|  | Deep Squats | 1:45 | 45Hz | High |  |
|  | Dynamic squats | 2:45 | 45Hz | High |  |
|  | Core strength exercises | 1:45 | 45Hz | Low | 20:45 minutes |
|  | Balanced | 1:45 | 45Hz | High |  |
|  | Abdominal exercises | 1:45 | 45Hz | Low |  |
|  | Right leg exercises | 1:45 | 45Hz | Low |  |
|  | Left leg exercises | 1:45 | 45Hz | Low |  |
|  | Chair squat | 1:45 | 45Hz | Low |  |
|  | Stretching exercises | 1:60 | 50Hz | Low |  |
|  | Relaxation exercises | 1:60 | 50Hz | Low |  |
|  |  |  |  |  |  |
| **WEEKS(22-26)- AUGMENTATION OF TIMING FOR DYNAMIC EXERCISES- REST TIME BETWEEN EXERCISES- 60 SECONDS** | | | | | |
|  |  |  |  |  |  |
|  | Light squats | 1:60 | 35Hz | Low |  |
|  | Deep Squats | 1:60 | 45Hz | High |  |
|  | Dynamic squats | 2:60 | 45Hz | High |  |
|  | Balanced | 1:60 | 45Hz | High |  |
|  | Core strength exercises | 1:60 | 45Hz | High | 21 minutes |
|  | Dynamic right leg exercises | 1:60 | 45Hz | Low |  |
|  | Dynamic left leg exercises | 1:60 | 45Hz | Low |  |
|  | Abdominal exercises | 1:60 | 45Hz | Low |  |
|  | Stretching exercises | 1:60 | 50Hz | Low |  |
|  | Relaxation exercises | 1:60 | 50Hz | Low |  |
|  |  |  |  |  |  |
| **WEEKS(27-30)- DYNAMIC EXERCISES- REST TIME BETWEEN EXERCISES- 30 SECONDS** | | | | | |
|  |  |  |  |  |  |
|  | Light squats | 1:60 | 35Hz | Low |  |
|  | Deep Squats | 1:60 | 45Hz | High |  |
|  | Dynamic squats | 2:60 | 45Hz | High |  |
|  | Balanced | 1:60 | 45Hz | High |  |
|  | Abdominal exercises | 1:60 | 45Hz | Low | 21 minutes |
|  | Chair squat | 1:60 | 45Hz | High |  |
|  | Right leg exercises | 1:60 | 45Hz | Low |  |
|  | Left leg exercises | 1:60 | 45Hz | Low |  |
|  | Stretching exercises | 1:60 | 50Hz | Low |  |
|  | Relaxation exercises | 1:60 | 50Hz | Low |  |
|  |  |  |  |  |  |
| **WEEK(31-33)- FREQUENCY VARIATION- REST TIME BETWEEN EXERCISES- 60 SECONDS** | | | | | |
|  |  |  |  |  |  |
|  | Light squats | 1:45 | 35Hz | Low |  |
|  | Deep Squats | 1:45 | 30-40Hz | High |  |
|  | Dynamic squats | 1:45 | 30-40Hz | High |  |
|  | Core strength exercises | 1:45 | 40Hz | High |  |
|  | Right leg exercises | 1:45 | 40Hz | Low |  |
|  | Left leg exercises | 1:45 | 40Hz | Low | 18 minutes |
|  | Abdominal exercises | 1:45 | 40Hz | Low |  |
|  | Chair squat | 1:45 | 30-40Hz | High |  |
|  | Stretching exercises | 1:60 | 50Hz | Low |  |
|  | Relaxation exercises | 1:60 | 50Hz | Low |  |
|  |  |  |  |  |  |
| **WEEKS(34-37) - FREQUENCY VARIATION- REST TIME BETWEEN EXERCISES- 60 SECONDS** | | | | | |
|  |  |  |  |  |  |
|  | Light squats | 1:60 | 30Hz | Low |  |
|  | Deep Squats | 1:60 | 30-40Hz | High |  |
|  | Balanced | 1:60 | 30-40Hz | High |  |
|  | Dynamic squats | 2:60 | 45Hz | High | 19 minutes |
|  | Core strength exercises | 1:60 | 45Hz | High |  |
|  | Right leg exercises | 1:60 | 45Hz | Low |  |
|  | Left leg exercises | 1:60 | 45Hz | Low |  |
|  | Stretching exercises | 1:60 | 50Hz | Low |  |
|  | Relaxation exercises | 1:60 | 50Hz | Low |  |
|  |  |  |  |  |  |
| **WEEKS(38-40)- FREQUENCY VARIATION- REST TIME BETWEEN EXERCISES- 60 SECONDS** | | | | | |
|  |  |  |  |  |  |
|  | Light squats | 1:60 | 30Hz | Low |  |
|  | Deep Squats | 1:60 | 30-40Hz | High |  |
|  | Dynamic squats | 2:60 | 30-40Hz | High |  |
|  | Balanced | 1:60 | 30-40Hz | Low |  |
|  | Right leg exercises | 1:60 | 30-40Hz | Low | 21 minutes |
|  | Left leg exercises | 1:60 | 30-40Hz | Low |  |
|  | Core strength exercises | 1:60 | 30-40Hz | Low |  |
|  | Chair squat | 1:60 | 30-40Hz | Low |  |
|  | Stretching exercises | 1:60 | 50Hz | Low |  |
|  | Relaxation exercises | 1:60 | 50Hz | Low |  |
|  |  |  |  |  |  |
| **WEEKS(41-END) - FREQUENCY VARIATION- REST TIME BETWEEN EXERCISES- 60 SECONDS** | | | | | |
|  |  |  |  |  |  |
|  | Light squats | 1:60 | 30hz | Low |  |
|  | Deep squats | 1:60 | 30-40hz | High |  |
|  | Dynamic squats | 2:60 | 30-40hz | High |  |
|  | Right leg exercises | 1:60 | 30-40hz | High |  |
|  | Left leg exercises | 1:60 | 30-40hz | High | 21 minutes |
|  | Balanced | 1:60 | 30-40hz | High |  |
|  | Core strength exercises | 1:60 | 30-40hz | High |  |
|  | Abdominal exercises | 1:60 | 30-40hz | Low |  |
|  | Stretching exercises | 1:60 | 50hz | Low |  |
|  | Relaxation exercises | 1:60 | 50hz | Low |  |

**SUPPLEMENTAL DATA 3:** Fardellone self-questionnaire

The purpose of this questionnaire is to estimate the amount of calcium in your diet. Please write down your answers or circle the correct answer where necessary. Thank you.

**Daily calcium content**

**1-** Do you drink milk every day? 1-

- If so, how much do you drink per day?

Number of glasses |___|

Number of cups|___|

Number of bowls|___|

- If not, how much do you drink per week?

Number of glasses||

Number of cups|___|

Number of bowls|___|

**2-** Do you eat yoghurt or dessert cream? 2-

- If so, how many per week?

**3-** Do you eat cottage cheese? If yes: 3-

- - How many 100g jars per week?
  - How many 500g pots per week?
  - How many 1kg pots per week?

**4-** Do you eat small Swiss cheeses? If yes : 4-

- How many "small sizes" per week?
- How many "big sizes" per week?

**5-** Do you eat Gruyere, Edam, Gouda, ...or any other hard cheese? 5-

If yes :

- How much do you eat per week?
- Are your portions **small medium large**

**6-Do you eat** camembert, brie, chèvre... or another soft cheese? 6-

If yes :

- How much do you eat per week?

Are your portions **small medium large**

**7-** Do you eat meat or fish (including ham and cold cuts) every day? 7-

the days?

- If so, how many times a day?

Are your portions **small medium large**

- If not, how many times a week?

Are your portions **small medium large**

**8-** How many eggs do you eat on average per week? 8-

**9-** How often do you eat potatoes (steamed, boiled...) 9-

per week ?

Are your portions **small medium large**

**10-** How often do you eat Chips per week? 10-

Are your portions **small medium large**

**11-** How often do you eat pasta or semolina per week? 11-

Are your portions **small medium large**

**12-** How often do you eat pulses (lentils, beans)? 12-

chickpeas...) per week ?

Are your portions **small medium large**

**13-** How often do you eat green vegetables (including soup) 13-

per week ?

Are your portions **small medium large**

**14-** How much bread do you eat on average per day? 14-

ficelles per day |___|

baguettes per day |___|

rusks per day |___|

**15-** How much fruit do you eat per week? 15-

**16-** Do you eat milk (or white) chocolate during the week? 16-

- If so, how much do you eat per week?

Number of bars per week |___|

Number of slabs per week |___|

**17-** Do you eat dark chocolate during the week? 17-

- If so, how much do you eat per week?

Number of bars per week |___|

Number of slabs per week. |___|

**18-** How much tap water do you drink per day? 18-

Number of drinks per day?

Number of litres per day?

**19-** Do you drink mineral water? 19-

- If yes : Number of drinks per day |___| Number of litres per day |___|
- Which one do you drink most often?

**Badoit Contrex Evian Perrier**

**Vichy Vittel GS*Vittel H**Other**

Great spring - ** Hepar

**20-** Do you drink wine, beer, cider or fruit juice every day? 20-

- If yes, how many drinks per day?

**Daily Calcium Ration, TOTAL* :**

**SUPPLEMENTAL DATA 4:** : Descriptive statistics for DXA and 3DShaper parameters

|  | **Group** | | | | | | | | | | | | | | | |
| --- | --- | --- | --- | --- | --- | --- | --- | --- | --- | --- | --- | --- | --- | --- | --- | --- |
|  | **Control** | | | | | | | | **Vibration** | | | | | | | |
|  | **Month** | | | | | | | | **Month** | | | | | | | |
|  | **Baseline** | | **Month 6** | | **Month 12** | | **Month 18** | | **Baseline** | | **Month 6** | | **Month 12** | | **Month 18** | |
|  | **Mean** | **SD** | **Mean** | **SD** | **Mean** | **SD** | **Mean** | **SD** | **Mean** | **SD** | **Mean** | **SD** | **Mean** | **SD** | **Mean** | **SD** |
| **Total BMD (g/cm^2^)** | 1.02 | 0.09 | 1.02 | 0.1 | 1.01 | 0.09 | 1.01 | 0.1 | 1.01 | 0.1 | 1.01 | 0.1 | 1. | 0.1 | 1. | 0.1 |
| **Total T-score (SD)** | 0.05 | 0.93 | -0.01 | 0.95 | -0.06 | 0.94 | -0.07 | 0.97 | -0.08 | 0.98 | -0.11 | 0.99 | -0.15 | 0.99 | -0.18 | 0.99 |
| **Bone mass (g)** | 2101.52 | 260.24 | 2095.16 | 263.33 | 2083.96 | 262.9 | 2075.48 | 256.6 | 2058.79 | 253.65 | 2055.11 | 253.15 | 2050.69 | 253.45 | 2042.67 | 254.13 |
| **Fat mass (g)** | 26845.52 | 9543.16 | 27214.33 | 9993.51 | 27792.64 | 10656.03 | 27462.37 | 10049.97 | 24673.83 | 7314.66 | 24730.58 | 7108.03 | 24844.68 | 7414.64 | 25228.18 | 7325.82 |
| **Lean mass (g)** | 37858.96 | 4363.89 | 38381.82 | 4357.63 | 38737.75 | 4353.1 | 38618.58 | 4322.97 | 36812.57 | 4169.27 | 36981.51 | 4043.62 | 37172.2 | 4234.16 | 37289.32 | 4265.3 |
| **Body mass (g)** | 66806. | 13157.84 | 67691.31 | 13651.94 | 68614.35 | 14294.6 | 68156.42 | 13560.58 | 63545.19 | 10713.35 | 63767.21 | 10431.21 | 64067.57 | 10906.5 | 64560.18 | 10871.42 |
| **BMD spine (g/cm^2^)** | 1.06 | 0.16 | 1.05 | 0.16 | 1.05 | 0.17 | 1.05 | 0.17 | 1.04 | 0.15 | 1.03 | 0.15 | 1.03 | 0.15 | 1.02 | 0.15 |
| **T-score spine (SD)** | -0.86 | 1.35 | -0.92 | 1.33 | -0.95 | 1.38 | -0.92 | 1.39 | -1.01 | 1.21 | -1.07 | 1.22 | -1.12 | 1.21 | -1.13 | 1.24 |
| **Trabecular bone score L1-L4 (g/cm^2^)** | 1.26 | 0.09 | 1.26 | 0.09 | 1.25 | 0.09 | 1.25 | 0.09 | 1.28 | 0.1 | 1.29 | 0.1 | 1.27 | 0.1 | 1.26 | 0.1 |
| **BMD femoral neck (g/cm^2^)** | 0.85 | 0.09 | 0.85 | 0.09 | 0.85 | 0.09 | 0.85 | 0.09 | 0.84 | 0.09 | 0.83 | 0.09 | 0.83 | 0.09 | 0.83 | 0.1 |
| **T-score femoral neck (SD)** | -1.07 | 0.73 | -1.06 | 0.75 | -1.08 | 0.74 | -1.1 | 0.72 | -1.2 | 0.79 | -1.23 | 0.78 | -1.23 | 0.76 | -1.27 | 0.8 |
| **BMD Total Hip (g/cm^2^)** | 0.89 | 0.1 | 0.89 | 0.11 | 0.89 | 0.1 | 0.89 | 0.11 | 0.87 | 0.11 | 0.87 | 0.1 | 0.86 | 0.1 | 0.86 | 0.11 |
| **T-score total hip (SD)** | -0.88 | 0.85 | -0.91 | 0.88 | -0.93 | 0.87 | -0.95 | 0.89 | -1.11 | 0.88 | -1.12 | 0.87 | -1.13 | 0.87 | -1.17 | 0.89 |
| **3D-Shaper total cortical vBMD (mg/cm^3^)** | 803.9 | 84.05 | 800.65 | 87.49 | 800.77 | 83.51 | 798.19 | 85.09 | 788.19 | 84.62 | 786.84 | 84.01 | 789.34 | 87.35 | 781.26 | 82.01 |
| **3D-Shaper total trabecular vBMD (mg/cm^3^)** | 140.42 | 33.09 | 139.27 | 34.56 | 137.71 | 33.79 | 136.75 | 34.02 | 135.05 | 35.21 | 135.88 | 35.97 | 134.58 | 35.5 | 132.13 | 37.06 |
| **3D-Shaper cortical vBMD of the neck (mg/cm^3^)** | 803.7 | 74.97 | 802.15 | 77.39 | 803.29 | 74.88 | 799.21 | 76.64 | 786.53 | 74.77 | 785.1 | 73.03 | 787.66 | 76.28 | 780.78 | 72.91 |
| **3D-Shaper trabecular vBMD of the neck (mg/cm^3^)** | 187.91 | 37.06 | 187.18 | 39.13 | 185.03 | 39.87 | 184.94 | 38.2 | 185.99 | 37.51 | 186.32 | 39.25 | 185.04 | 38.44 | 182.2 | 39.99 |

**SUPPLEMENTAL DATA 5:** : Descriptive statistics for HR-pQCT Tibia

|  | **Group** | | | | | | | | | | | | | | | |
| --- | --- | --- | --- | --- | --- | --- | --- | --- | --- | --- | --- | --- | --- | --- | --- | --- |
|  | **Control** | | | | | | | | **Vibration** | | | | | | | |
|  | **Month** | | | | | | | | **Month** | | | | | | | |
|  | **Baseline** | | **Month 6** | | **Month 12** | | **Month 18** | | **Baseline** | | **Month 6** | | **Month 12** | | **Month 18** | |
|  | **Mean** | **SD** | **Mean** | **SD** | **Mean** | **SD** | **Mean** | **SD** | **Mean** | **SD** | **Mean** | **SD** | **Mean** | **SD** | **Mean** | **SD** |
| **Total BMD (mgHA/cm^3^)** | 257.5 | 49.15 | 257.6 | 49.32 | 255.66 | 49.82 | 255.31 | 49.45 | 253.4 | 48.44 | 252.39 | 47.83 | 252.05 | 47.47 | 250.21 | 47.63 |
| **Trabecular vBMD (mgHA/cm^3^)** | 154.53 | 34.96 | 154.79 | 34.8 | 153.7 | 34.72 | 153.76 | 34.65 | 152.4 | 35.93 | 152.48 | 35.5 | 152.69 | 35.74 | 152.2 | 36.03 |
| **BV/TV (%)** | 0.13 | 0.03 | 0.13 | 0.03 | 0.13 | 0.03 | 0.13 | 0.03 | 0.13 | 0.03 | 0.13 | 0.03 | 0.13 | 0.03 | 0.13 | 0.03 |
| **Trabecular inhomogeneity (Tb.1/N.SD; mm)** | 0.29 | 0.16 | 0.29 | 0.18 | 0.29 | 0.2 | 0.29 | 0.19 | 0.35 | 0.38 | 0.36 | 0.4 | 0.37 | 0.44 | 0.36 | 0.42 |
| **Cortical area (mm^2^)** | 96.6 | 14.51 | 96.96 | 14.76 | 96.29 | 15.28 | 97.07 | 14.94 | 92.39 | 15.72 | 92.52 | 15.72 | 92.66 | 15.71 | 92.18 | 15.83 |
| **Cortical vBMD (mgHA/cm^3^)** | 795.6 | 60.3 | 791.69 | 60.87 | 789.52 | 60.02 | 785.79 | 61.15 | 806.31 | 64.9 | 800.27 | 65.81 | 796.83 | 65.12 | 792.79 | 63.98 |
| **Cortical perimeter (mm)** | 99.49 | 7.38 | 99.33 | 7.33 | 100.28 | 10.71 | 99.23 | 7.47 | 99.86 | 6.7 | 99.71 | 6.68 | 99.44 | 6.62 | 99.45 | 6.57 |
| **Cortical thickness (mm)** | 1.07 | 0.18 | 1.07 | 0.19 | 1.07 | 0.19 | 1.07 | 0.19 | 1.02 | 0.18 | 1.02 | 0.18 | 1.03 | 0.18 | 1.02 | 0.18 |
| **Cortical porosity (%)** | 0.08 | 0.03 | 0.08 | 0.04 | 0.09 | 0.03 | 0.09 | 0.04 | 0.07 | 0.03 | 0.07 | 0.03 | 0.08 | 0.03 | 0.08 | 0.03 |
| **Ultimate load (N)** | -8028.69 | 1265.94 | -7998.63 | 1297.79 | -7953.9 | 1297.94 | -7966.98 | 1287.58 | -8020.71 | 1320.72 | -7950.34 | 1268.8 | -7932.25 | 1211.52 | -7888.73 | 1275.06 |
| **Trabecular load vs whole bone load (distal)** | 0.52 | 0.09 | 0.52 | 0.09 | 0.52 | 0.09 | 0.52 | 0.09 | 0.53 | 0.1 | 0.53 | 0.1 | 0.52 | 0.11 | 0.53 | 0.1 |
| **Trabecular load vs whole bone load (proximal)** | 0.32 | 0.08 | 0.31 | 0.08 | 0.32 | 0.09 | 0.32 | 0.09 | 0.33 | 0.09 | 0.33 | 0.09 | 0.32 | 0.09 | 0.33 | 0.09 |
| **Trabecular Von Mises stress (MPa)** | 52.76 | 7.11 | 52.29 | 7.12 | 52.38 | 7.07 | 52.27 | 7.07 | 54.06 | 7.18 | 53.49 | 6.35 | 52.71 | 7.46 | 53.04 | 6.36 |
| **Cortical Von Mises stress (MPa)** | 86.01 | 2.21 | 85.88 | 2.35 | 85.78 | 2.34 | 85.87 | 2.3 | 86.19 | 2.41 | 86.01 | 2.57 | 86.19 | 2.36 | 85.96 | 2.69 |
| **Total Volume (mm^3^)** | 19625.05 | 4851.78 | 19662.39 | 5146.32 | 19791.45 | 5408.48 | 19507.13 | 5063.04 | 18770.96 | 3965.69 | 18655.73 | 4045.49 | 18604.33 | 3990.16 | 18514.03 | 4088.33 |
| **MV/TV** | 0.55 | 0.15 | 0.55 | 0.15 | 0.54 | 0.15 | 0.55 | 0.15 | 0.57 | 0.13 | 0.57 | 0.13 | 0.57 | 0.13 | 0.57 | 0.14 |

**SUPPLEMENTAL DATA 6:** Descriptive statistics for HR-pQCT Radius

|  | **Group** | | | | | | | | | | | | | | | |
| --- | --- | --- | --- | --- | --- | --- | --- | --- | --- | --- | --- | --- | --- | --- | --- | --- |
|  | **Control** | | | | | | | | **Vibration** | | | | | | | |
|  | **Month** | | | | | | | | **Month** | | | | | | | |
|  | **Baseline** | | **Month 6** | | **Month 12** | | **Month 18** | | **Baseline** | | **Month 6** | | **Month 12** | | **Month 18** | |
|  | **Mean** | **SD** | **Mean** | **SD** | **Mean** | **SD** | **Mean** | **SD** | **Mean** | **SD** | **Mean** | **SD** | **Mean** | **SD** | **Mean** | **SD** |
| **Total BMD (mgHA/cm^3^)** | 295.95 | 67.08 | 296.46 | 67.2 | 294.02 | 66.61 | 292.24 | 69.76 | 295.05 | 59.35 | 294.1 | 58.87 | 293.32 | 59.23 | 290.96 | 58.15 |
| **Trabecular vBMD (mgHA/cm^3^)** | 149.62 | 39.62 | 149.66 | 39.14 | 148.87 | 39.7 | 147.34 | 40.91 | 142.75 | 41.17 | 142.85 | 41.01 | 143. | 41.77 | 141.97 | 41.71 |
| **BV/TV (%)** | 0.12 | 0.03 | 0.12 | 0.03 | 0.12 | 0.03 | 0.12 | 0.03 | 0.12 | 0.03 | 0.12 | 0.03 | 0.12 | 0.03 | 0.12 | 0.03 |
| **Trabecular inhomogeneity (Tb.1/N.SD; mm)** | 0.29 | 0.21 | 0.3 | 0.22 | 0.3 | 0.22 | 0.3 | 0.22 | 0.33 | 0.22 | 0.34 | 0.25 | 0.34 | 0.24 | 0.34 | 0.25 |
| **Cortical area (mm^2^)** | 48.88 | 8.26 | 49.24 | 8.14 | 48.86 | 8.29 | 50.39 | 15.56 | 48.73 | 7.64 | 48.55 | 7.74 | 48.6 | 7.57 | 48.18 | 7.58 |
| **Cortical vBMD (mgHA/cm^3^)** | 847. | 56.1 | 846.07 | 56.86 | 843.13 | 56.44 | 835.3 | 77.32 | 863.29 | 59.37 | 859.74 | 59.14 | 856.39 | 56.13 | 855.64 | 55.86 |
| **Cortical perimeter (mm)** | 68.8 | 10.67 | 67.43 | 6.15 | 67.31 | 6.25 | 67.26 | 6.12 | 66.76 | 5.04 | 66.68 | 4.95 | 66.52 | 4.94 | 66.39 | 4.85 |
| **Cortical thickness (mm)** | 0.81 | 0.16 | 0.81 | 0.16 | 0.81 | 0.16 | 0.82 | 0.21 | 0.82 | 0.15 | 0.81 | 0.15 | 0.82 | 0.15 | 0.81 | 0.15 |
| **Cortical porosity (%)** | 0.02 | 0.01 | 0.03 | 0.01 | 0.03 | 0.01 | 0.04 | 0.08 | 0.02 | 0.01 | 0.02 | 0.01 | 0.02 | 0.01 | 0.02 | 0.01 |
| **Ultimate load (N)** | -2859.75 | 551.01 | -2841.58 | 532.52 | -2779.68 | 531.53 | -2820.25 | 580.13 | -2901.77 | 541.36 | -2849.36 | 621. | -2837.69 | 547.62 | -2814.81 | 530.03 |
| **Trabecular load vs whole bone load (distal)** | 0.44 | 0.07 | 0.44 | 0.08 | 0.43 | 0.07 | 0.45 | 0.08 | 0.44 | 0.1 | 0.45 | 0.1 | 0.43 | 0.09 | 0.44 | 0.1 |
| **Trabecular load vs whole bone load (proximal)** | 0.16 | 0.06 | 0.16 | 0.06 | 0.15 | 0.05 | 0.16 | 0.05 | 0.16 | 0.06 | 0.16 | 0.07 | 0.15 | 0.06 | 0.16 | 0.06 |
| **Trabecular Von Mises stress (MPa)** | 42.42 | 6.29 | 42.28 | 6.27 | 40.98 | 6.49 | 42.44 | 6.75 | 43.61 | 7.24 | 43.34 | 8.33 | 42.53 | 7.25 | 42.98 | 7.47 |
| **Cortical Von Mises stress (MPa)** | 78.92 | 3.81 | 78.58 | 3.79 | 78.22 | 3.88 | 78.01 | 5.18 | 79.99 | 3.55 | 78.84 | 8.24 | 79.59 | 3.61 | 79.29 | 3.74 |

**SUPPLEMENTAL DATA 7:** Descriptive statistics for serum parameters

|  | **Group** | | | | | | | | | | | | | | | |
| --- | --- | --- | --- | --- | --- | --- | --- | --- | --- | --- | --- | --- | --- | --- | --- | --- |
|  | **Control** | | | | | | | | **Vibration** | | | | | | | |
|  | **Month** | | | | | | | | **Month** | | | | | | | |
|  | **Baseline** | | **Month 6** | | **Month 12** | | **Month 18** | | **Baseline** | | **Month 6** | | **Month 12** | | **Month 18** | |
|  | **Mean** | **SD** | **Mean** | **SD** | **Mean** | **SD** | **Mean** | **SD** | **Mean** | **SD** | **Mean** | **SD** | **Mean** | **SD** | **Mean** | **SD** |
| **P1NP (µg/L)** | 55.77 | 23.87 | 56. | 22.49 | 55.34 | 22.75 | 55.16 | 22.57 | 56.17 | 18.39 | 55.4 | 19.1 | 57.22 | 19.47 | 54.14 | 17.78 |
| **CTX (pmol/L)** | 4141.05 | 2379.15 | 4364.39 | 2470.48 | 4166.47 | 2243.03 | 4442.14 | 2413.61 | 4332.82 | 1836.07 | 4546.18 | 2127.92 | 4839.11 | 1992.26 | 5137.39 | 2173.57 |
| **BAP (µg/L)** | 12.25 | 4.12 | 13.54 | 4.46 | 14.61 | 5.21 | 15.73 | 5.76 | 11.71 | 3.6 | 12.92 | 3.73 | 14.22 | 4.25 | 14.58 | 4.34 |
| **Sclerostin (ng/mL)** | 0.64 | 0.15 | 0.65 | 0.16 | 0.64 | 0.16 | 0.65 | 0.16 | 0.6 | 0.15 | 0.59 | 0.14 | 0.6 | 0.14 | 0.6 | 0.17 |

**SUPPLEMENTAL DATA 8:** : Descriptive statistics for DXA and 3DShaper parameters with age groups (≤64&>64)

|  | **Group** | | | | | | | | | | | | | | | | | | | | | | | | | | | | | | | |
| --- | --- | --- | --- | --- | --- | --- | --- | --- | --- | --- | --- | --- | --- | --- | --- | --- | --- | --- | --- | --- | --- | --- | --- | --- | --- | --- | --- | --- | --- | --- | --- | --- |
|  | **Control** | | | | | | | | | | | | | | | | **Vibration** | | | | | | | | | | | | | | | |
|  | **Age groups** | | | | | | | | | | | | | | | | **Age groups** | | | | | | | | | | | | | | | |
|  | **Less than ≤64** | | | | | | | | **Greater than 64** | | | | | | | | **Less than ≤ 64** | | | | | | | | **Greater than 64.00** | | | | | | | |
|  | **Month** | | | | | | | | **Month** | | | | | | | | **Month** | | | | | | | | **Month** | | | | | | | |
|  | **Baseline** | | **Month 6** | | **Month 12** | | **Month 18** | | **Baseline** | | **Month 6** | | **Month 12** | | **Month 18** | | **Baseline** | | **Month 6** | | **Month 12** | | **Month 18** | | **Baseline** | | **Month 6** | | **Month 12** | | **Month 18** | |
|  | **Mean** | **SD** | **Mean** | **SD** | **Mean** | **SD** | **Mean** | **SD** | **Mean** | **SD** | **Mean** | **SD** | **Mean** | **SD** | **Mean** | **SD** | **Mean** | **SD** | **Mean** | **SD** | **Mean** | **SD** | **Mean** | **SD** | **Mean** | **SD** | **Mean** | **SD** | **Mean** | **SD** | **Mean** | **SD** |
| **Total BMD (g/cm^2^)** | 1.01 | 0.10 | 0.99 | 0.09 | 1.00 | 0.09 | 1.00 | 0.09 | 1.03 | 0.09 | 1.03 | 0.10 | 1.02 | 0.10 | 1.02 | 0.10 | 1.01 | 0.10 | 1.01 | 0.10 | 1.01 | 0.10 | 1.00 | 0.11 | 1.02 | 0.09 | 1.01 | 0.10 | 1.00 | 0.10 | 1.00 | 0.10 |
| **Total T-score (SD)** | -0.07 | 0.98 | -0.25 | 0.89 | -0.24 | 0.87 | -0.23 | 0.90 | 0.11 | 0.90 | 0.11 | 0.96 | 0.01 | 0.96 | -0.02 | 0.99 | -0.12 | 1.02 | -0.14 | 1.02 | -0.14 | 1.03 | -0.18 | 1.05 | 0.00 | 0.92 | -0.08 | 0.98 | -0.16 | 0.95 | -0.18 | 0.94 |
| **Bone mass (g)** | 2057.29 | 253.61 | 2022.06 | 236.19 | 2016.46 | 233.99 | 2009.68 | 243.72 | 2126.09 | 262.62 | 2130.61 | 270.13 | 2110.96 | 270.47 | 2096.82 | 258.63 | 2040.04 | 242.46 | 2033.28 | 232.05 | 2035.73 | 236.86 | 2028.30 | 243.88 | 2087.62 | 270.62 | 2077.39 | 273.63 | 2064.78 | 269.73 | 2054.65 | 264.03 |
| **Fat mass (g)** | 24579.99 | 8930.01 | 24800.54 | 9818.91 | 25249.88 | 10779.18 | 25180.47 | 9600.82 | 28104.15 | 9708.00 | 28384.65 | 9939.70 | 28809.75 | 10511.44 | 28202.44 | 10143.92 | 23966.12 | 7643.39 | 24172.40 | 7099.54 | 24417.45 | 7545.24 | 24649.27 | 7315.76 | 25762.61 | 6728.32 | 25300.16 | 7144.50 | 25246.77 | 7341.65 | 25710.61 | 7367.54 |
| **Lean mass (g)** | 37217.92 | 3840.61 | 37674.94 | 3877.88 | 37890.72 | 4176.79 | 38022.43 | 4511.84 | 38215.09 | 4619.99 | 38724.55 | 4560.71 | 39076.56 | 4405.11 | 38811.92 | 4273.60 | 36755.81 | 3960.80 | 36797.13 | 3591.83 | 36949.07 | 3570.78 | 36878.48 | 3811.82 | 36899.89 | 4523.15 | 37169.66 | 4487.91 | 37382.20 | 4802.17 | 37631.70 | 4616.73 |
| **Body mass (g)** | 63855.21 | 12118.38 | 64497.55 | 12871.95 | 65157.06 | 14074.63 | 65212.57 | 13199.44 | 68445.33 | 13516.04 | 69239.81 | 13843.89 | 69997.27 | 14245.99 | 69111.19 | 13626.44 | 62761.98 | 10704.48 | 63002.80 | 9913.36 | 63402.25 | 10375.94 | 63556.05 | 10363.39 | 64750.13 | 10753.26 | 64547.21 | 10981.90 | 64693.75 | 11450.76 | 65396.96 | 11305.05 |
| **BMD spine (g/cm^2^)** | 1.02 | 0.16 | 0.99 | 0.13 | 0.98 | 0.15 | 0.98 | 0.15 | 1.07 | 0.16 | 1.08 | 0.17 | 1.07 | 0.17 | 1.07 | 0.17 | 1.02 | 0.14 | 1.01 | 0.13 | 1.01 | 0.14 | 1.01 | 0.14 | 1.07 | 0.16 | 1.05 | 0.16 | 1.04 | 0.15 | 1.04 | 0.16 |
| **T-score spine (SD)** | -1.14 | 1.35 | -1.41 | 1.07 | -1.46 | 1.25 | -1.47 | 1.29 | -0.71 | 1.33 | -0.68 | 1.38 | -0.75 | 1.38 | -0.74 | 1.39 | -1.17 | 1.13 | -1.25 | 1.09 | -1.21 | 1.15 | -1.28 | 1.15 | -0.77 | 1.31 | -0.89 | 1.33 | -1.03 | 1.28 | -1.01 | 1.31 |
| **Trabecular bone score L1-L4 (g/cm^2^)** | 1.26 | 0.09 | 1.26 | 0.08 | 1.25 | 0.09 | 1.24 | 0.08 | 1.25 | 0.09 | 1.26 | 0.09 | 1.25 | 0.09 | 1.25 | 0.09 | 1.29 | 0.11 | 1.29 | 0.10 | 1.28 | 0.10 | 1.27 | 0.11 | 1.25 | 0.10 | 1.28 | 0.10 | 1.26 | 0.10 | 1.25 | 0.10 |
| **BMD femoral neck (g/cm^2^)** | 0.84 | 0.09 | 0.85 | 0.10 | 0.85 | 0.10 | 0.83 | 0.10 | 0.86 | 0.08 | 0.86 | 0.08 | 0.85 | 0.08 | 0.85 | 0.08 | 0.82 | 0.09 | 0.82 | 0.10 | 0.82 | 0.09 | 0.82 | 0.10 | 0.86 | 0.10 | 0.84 | 0.09 | 0.84 | 0.09 | 0.84 | 0.09 |
| **T-score femoral neck (SD)** | -1.14 | 0.78 | -1.12 | 0.85 | -1.07 | 0.86 | -1.24 | 0.82 | -1.04 | 0.70 | -1.03 | 0.70 | -1.09 | 0.69 | -1.06 | 0.69 | -1.30 | 0.76 | -1.31 | 0.79 | -1.32 | 0.79 | -1.35 | 0.85 | -1.04 | 0.82 | -1.14 | 0.77 | -1.16 | 0.74 | -1.20 | 0.75 |
| **BMD total hip (g/cm^2^)** | 0.87 | 0.10 | 0.86 | 0.11 | 0.87 | 0.11 | 0.86 | 0.11 | 0.91 | 0.10 | 0.91 | 0.10 | 0.89 | 0.10 | 0.89 | 0.10 | 0.85 | 0.11 | 0.85 | 0.11 | 0.85 | 0.11 | 0.84 | 0.12 | 0.89 | 0.10 | 0.88 | 0.10 | 0.88 | 0.10 | 0.87 | 0.10 |
| **T-score total hip (SD)** | -1.09 | 0.87 | -1.16 | 0.89 | -1.06 | 0.91 | -1.14 | 0.95 | -0.77 | 0.83 | -0.79 | 0.86 | -0.88 | 0.86 | -0.88 | 0.86 | -1.24 | 0.90 | -1.26 | 0.93 | -1.24 | 0.93 | -1.29 | 0.98 | -0.90 | 0.82 | -0.97 | 0.80 | -1.03 | 0.81 | -1.07 | 0.80 |
| **3DShaper total cortical vBMD (mg/cm^3^)** | 787.94 | 86.66 | 783.37 | 88.44 | 791.59 | 84.56 | 790.17 | 95.24 | 812.77 | 81.92 | 809.03 | 86.46 | 804.44 | 83.42 | 800.80 | 82.07 | 780.54 | 80.39 | 775.54 | 84.60 | 782.37 | 87.29 | 776.00 | 80.33 | 799.95 | 90.55 | 798.37 | 82.68 | 795.89 | 87.76 | 785.63 | 83.88 |
| **3DShaper total trabecular vBMD (mg/cm^3^)** | 135.60 | 34.72 | 133.23 | 36.25 | 135.67 | 36.56 | 132.45 | 38.27 | 143.09 | 32.12 | 142.20 | 33.59 | 138.53 | 32.85 | 138.14 | 32.69 | 130.61 | 38.14 | 131.87 | 39.35 | 131.73 | 39.38 | 129.37 | 40.97 | 141.89 | 29.32 | 139.97 | 32.06 | 137.27 | 31.58 | 134.43 | 33.67 |
| **3DShaper cortical vBMD of the neck (mg/cm^3^)** | 789.37 | 75.12 | 791.59 | 77.95 | 799.07 | 75.12 | 792.57 | 79.95 | 811.67 | 74.29 | 807.27 | 77.19 | 804.97 | 75.26 | 801.37 | 75.97 | 778.71 | 68.23 | 773.95 | 69.26 | 780.47 | 73.28 | 774.39 | 67.90 | 798.55 | 83.33 | 796.47 | 75.70 | 794.44 | 79.13 | 786.12 | 77.07 |
| **3DShaper trabecular vBMD of the neck (mg/cm^3^)** | 189.24 | 40.64 | 187.53 | 44.02 | 187.96 | 46.33 | 183.01 | 43.78 | 187.17 | 35.24 | 187.00 | 36.88 | 183.86 | 37.28 | 185.56 | 36.51 | 181.98 | 40.18 | 183.87 | 43.06 | 183.11 | 42.53 | 181.13 | 44.29 | 192.16 | 32.51 | 188.83 | 35.21 | 186.86 | 34.48 | 183.09 | 36.43 |

**SUPPLEMENTAL DATA 9:** : Descriptive statistics for HR-pQCT Tibia with age groups (≤64&>64)

|  | | **Group** | | | | | | | | | | | | | | | | | | | | | | | | | | | | | | | | | | | | | | | | | | | | | | | |
| --- | --- | --- | --- | --- | --- | --- | --- | --- | --- | --- | --- | --- | --- | --- | --- | --- | --- | --- | --- | --- | --- | --- | --- | --- | --- | --- | --- | --- | --- | --- | --- | --- | --- | --- | --- | --- | --- | --- | --- | --- | --- | --- | --- | --- | --- | --- | --- | --- | --- |
|  | | **Control** | | | | | | | | | | | | | | | | | | | | | | | | | **Vibration** | | | | | | | | | | | | | | | | | | | | | | |
|  | | **Age groups** | | | | | | | | | | | | | | | | | | | | | | | | | **Age groups** | | | | | | | | | | | | | | | | | | | | | | |
|  | | **Less than ≤64** | | | | | | | | | | | | | **Greater than 64** | | | | | | | | | | | | **Less than ≤ 64** | | | | | | | | | | | | **Greater than 64** | | | | | | | | | | |
|  | | **Month** | | | | | | | | | | | | | **Month** | | | | | | | | | | | | **Month** | | | | | | | | | | | | **Month** | | | | | | | | | | |
|  | | **Baseline** | | | | | **Month 6** | | | **Month 12** | | **Month 18** | | | **Baseline** | | | **Month 6** | | | **Month 12** | | | **Month 18** | | | **Baseline** | | | **Month 6** | | | **Month 12** | | | **Month 18** | | | **Baseline** | | | **Month 6** | | | **Month 12** | | | **Month 18** | |
|  | | **Mean** | | | **SD** | | **Mean** | | **SD** | **Mean** | **SD** | **Mean** | | **SD** | **Mean** | | **SD** | **Mean** | | **SD** | **Mean** | | **SD** | **Mean** | | **SD** | **Mean** | | **SD** | **Mean** | | **SD** | **Mean** | | **SD** | **Mean** | | **SD** | **Mean** | | **SD** | **Mean** | | **SD** | **Mean** | | **SD** | **Mean** | **SD** |
| **Total BMD (mgHA/cm^3^)** | | 255.52 | | | 61.06 | | 253.58 | | 61.97 | 255.48 | 62.34 | 252.52 | | 64.15 | 258.52 | | 42.28 | 259.58 | | 42.21 | 255.73 | | 44.48 | 256.24 | | 44.04 | 259.35 | | 54.40 | 258.37 | | 50.35 | 258.99 | | 50.79 | 256.54 | | 51.42 | 243.12 | | 34.31 | 245.85 | | 44.58 | 245.12 | | 43.35 | 244.68 | 43.84 |
| **Trabecular vBMD (mgHA/cm^3^)** | | 147.43 | | | 36.77 | | 146.42 | | 36.28 | 147.31 | 37.83 | 142.30 | | 34.09 | 158.20 | | 33.73 | 158.90 | | 33.60 | 156.24 | | 33.38 | 157.58 | | 34.24 | 152.90 | | 38.72 | 149.97 | | 35.63 | 150.92 | | 36.43 | 149.07 | | 37.77 | 151.52 | | 31.07 | 155.22 | | 35.57 | 154.45 | | 35.35 | 154.94 | 34.61 |
| **BV/TV (%)** | | 0.12 | | | 0.03 | | 0.12 | | 0.03 | 0.12 | 0.03 | 0.12 | | 0.03 | 0.13 | | 0.03 | 0.13 | | 0.03 | 0.13 | | 0.03 | 0.13 | | 0.03 | 0.13 | | 0.03 | 0.13 | | 0.03 | 0.13 | | 0.03 | 0.12 | | 0.03 | 0.13 | | 0.03 | 0.13 | | 0.03 | 0.13 | | 0.03 | 0.13 | 0.03 |
| **Trabecular inhomogeneity (Tb.1/N.SD; mm)** | | 0.32 | | | 0.21 | | 0.32 | | 0.24 | 0.34 | 0.31 | 0.34 | | 0.30 | 0.27 | | 0.13 | 0.27 | | 0.14 | 0.27 | | 0.13 | 0.28 | | 0.13 | 0.39 | | 0.47 | 0.37 | | 0.40 | 0.39 | | 0.44 | 0.39 | | 0.44 | 0.28 | | 0.07 | 0.34 | | 0.40 | 0.34 | | 0.44 | 0.33 | 0.41 |
| **Cortical area (mm^2^)** | | 94.73 | | | 12.80 | | 94.62 | | 13.38 | 93.97 | 9.26 | 94.62 | | 9.40 | 97.57 | | 15.34 | 98.11 | | 15.38 | 97.22 | | 17.07 | 97.89 | | 16.36 | 93.98 | | 15.56 | 94.48 | | 15.91 | 94.63 | | 15.68 | 94.95 | | 15.86 | 89.64 | | 15.85 | 90.37 | | 15.41 | 90.68 | | 15.67 | 89.76 | 15.57 |
| **Cortical vBMD (mgHA/cm^3^)** | | 819.52 | | | 59.25 | | 812.88 | | 60.32 | 817.59 | 60.83 | 817.08 | | 67.07 | 783.23 | | 57.50 | 781.28 | | 58.88 | 778.39 | | 56.38 | 775.36 | | 55.76 | 824.26 | | 64.19 | 826.31 | | 60.31 | 823.27 | | 59.05 | 821.95 | | 55.46 | 775.32 | | 54.22 | 771.80 | | 60.06 | 770.40 | | 60.51 | 767.28 | 60.37 |
| **Cortical perimeter (mm)** | | 99.58 | | | 7.81 | | 99.26 | | 7.82 | 99.01 | 8.29 | 98.80 | | 8.60 | 99.44 | | 7.22 | 99.37 | | 7.14 | 100.78 | | 11.56 | 99.37 | | 7.12 | 99.43 | | 6.22 | 99.18 | | 6.07 | 98.76 | | 6.13 | 98.92 | | 6.05 | 100.58 | | 7.52 | 100.29 | | 7.32 | 100.13 | | 7.08 | 99.91 | 7.03 |
| **Cortical thickness (mm)** | | 1.05 | | | 0.22 | | 1.05 | | 0.23 | 1.05 | 0.20 | 1.06 | | 0.21 | 1.08 | | 0.17 | 1.08 | | 0.17 | 1.07 | | 0.19 | 1.07 | | 0.18 | 1.04 | | 0.19 | 1.05 | | 0.20 | 1.05 | | 0.20 | 1.06 | | 0.19 | 0.99 | | 0.16 | 0.99 | | 0.16 | 1.00 | | 0.16 | 0.99 | 0.17 |
| **Cortical porosity (%)** | | 0.06 | | | 0.02 | | 0.06 | | 0.02 | 0.07 | 0.02 | 0.06 | | 0.02 | 0.09 | | 0.04 | 0.09 | | 0.04 | 0.09 | | 0.03 | 0.10 | | 0.04 | 0.07 | | 0.03 | 0.06 | | 0.03 | 0.07 | | 0.03 | 0.07 | | 0.02 | 0.08 | | 0.03 | 0.09 | | 0.04 | 0.09 | | 0.04 | 0.09 | 0.04 |
| **Ultimate load (N)** | | -7951.15 | | | 1041.40 | | -7781.38 | | 1066.08 | -7862.71 | 1050.46 | -7693.52 | | 1081.27 | -8068.81 | | 1374.59 | -8105.42 | | 1393.74 | -7990.08 | | 1390.00 | -8058.13 | | 1344.44 | -8126.93 | | 1355.63 | -8055.34 | | 1280.55 | -8050.25 | | 1241.12 | -8030.74 | | 1283.35 | -7837.23 | | 1257.18 | -7835.57 | | 1260.78 | -7816.87 | | 1184.33 | -7764.48 | 1268.10 |
| **Trabecular load vs whole bone load (distal)** | | 0.51 | | | 0.09 | | 0.49 | | 0.09 | 0.50 | 0.09 | 0.47 | | 0.08 | 0.53 | | 0.09 | 0.53 | | 0.09 | 0.53 | | 0.09 | 0.53 | | 0.09 | 0.53 | | 0.09 | 0.51 | | 0.09 | 0.51 | | 0.10 | 0.51 | | 0.10 | 0.54 | | 0.10 | 0.55 | | 0.10 | 0.53 | | 0.12 | 0.55 | 0.10 |
| **Trabecular load vs whole bone load (proximal)** | | 0.31 | | | 0.08 | | 0.29 | | 0.08 | 0.30 | 0.08 | 0.29 | | 0.06 | 0.33 | | 0.08 | 0.32 | | 0.08 | 0.32 | | 0.09 | 0.32 | | 0.09 | 0.33 | | 0.08 | 0.32 | | 0.08 | 0.32 | | 0.09 | 0.31 | | 0.09 | 0.34 | | 0.11 | 0.34 | | 0.10 | 0.33 | | 0.10 | 0.34 | 0.09 |
| **Trabecular Von Mises stress (MPa)** | | 52.61 | | | 7.37 | | 50.88 | | 7.19 | 52.06 | 7.58 | 50.66 | | 8.08 | 52.83 | | 7.04 | 52.98 | | 7.04 | 52.50 | | 6.91 | 52.81 | | 6.68 | 54.40 | | 7.44 | 53.68 | | 5.85 | 53.08 | | 5.99 | 53.21 | | 6.24 | 53.49 | | 6.80 | 53.29 | | 6.91 | 52.34 | | 8.71 | 52.89 | 6.54 |
| **Cortical Von Mises stress (MPa)** | | 86.94 | | | 1.88 | | 86.81 | | 1.96 | 86.97 | 2.10 | 87.01 | | 2.39 | 85.53 | | 2.23 | 85.42 | | 2.41 | 85.31 | | 2.28 | 85.49 | | 2.16 | 86.56 | | 2.37 | 86.79 | | 2.30 | 86.88 | | 2.04 | 86.92 | | 2.10 | 85.55 | | 2.37 | 85.16 | | 2.61 | 85.51 | | 2.48 | 85.13 | 2.89 |
| **Total_Volume (mm^3^)** | 18695.40 | | 4237.62 | 19413.96 | | 5448.84 | | 18530.66 | | 4678.41 | 18394.29 | | 4184.30 | 20105.90 | | 5109.09 | 19784.50 | | 5034.67 | 20291.76 | | 5628.38 | 19878.07 | | 5300.49 | 18034.28 | | 3740.74 | 17947.64 | | 3553.88 | 18092.56 | | 3275.18 | 17856.35 | | 3605.45 | 20021.08 | | 4078.77 | 19448.12 | | 4442.83 | 19127.73 | | 4588.31 | 19101.73 | | 4431.85 |
| **MV/TV** | 0.57 | | 0.16 | 0.57 | | 0.17 | | 0.58 | | 0.16 | 0.58 | | 0.16 | 0.54 | | 0.14 | 0.54 | | 0.14 | 0.53 | | 0.15 | 0.53 | | 0.14 | 0.58 | | 0.13 | 0.58 | | 0.12 | 0.57 | | 0.13 | 0.57 | | 0.14 | 0.55 | | 0.13 | 0.56 | | 0.14 | 0.57 | | 0.14 | 0.56 | | 0.14 |
|  | |  | | |  | |  | |  |  |  |  | |  |  | |  |  | |  |  | |  |  | |  |  | |  |  | |  |  | |  |  | |  |  | |  |  | |  |  | |  |  |  |

**SUPPLEMENTAL DATA 10:** Descriptive statistics for HR-pQCT Radius with age groups (≤64&>64)

|  | **Group** | | | | | | | | | | | | | | | | | | | | | | | | | | | | | | | |
| --- | --- | --- | --- | --- | --- | --- | --- | --- | --- | --- | --- | --- | --- | --- | --- | --- | --- | --- | --- | --- | --- | --- | --- | --- | --- | --- | --- | --- | --- | --- | --- | --- |
|  | **Control** | | | | | | | | | | | | | | | | **Vibration** | | | | | | | | | | | | | | | |
|  | **Age groups** | | | | | | | | | | | | | | | | **Age groups** | | | | | | | | | | | | | | | |
|  | **Less than ≤64** | | | | | | | | **Greater than 64** | | | | | | | | **Less than ≤64.00** | | | | | | | | **Greater than 64** | | | | | | | |
|  | **Month** | | | | | | | | **Month** | | | | | | | | **Month** | | | | | | | | **Month** | | | | | | | |
|  | **Baseline** | | **Month 6** | | **Month 12** | | **Month 18** | | **Baseline** | | **Month 6** | | **Month 12** | | **Month 18** | | **Baseline** | | **Month 6** | | **Month 12** | | **Month 18** | | **Baseline** | | **Month 6** | | **Month 12** | | **Month 18** | |
|  | **Mean** | **SD** | **Mean** | **SD** | **Mean** | **SD** | **Mean** | **SD** | **Mean** | **SD** | **Mean** | **SD** | **Mean** | **SD** | **Mean** | **SD** | **Mean** | **SD** | **Mean** | **SD** | **Mean** | **SD** | **Mean** | **SD** | **Mean** | **SD** | **Mean** | **SD** | **Mean** | **SD** | **Mean** | **SD** |
| **Total BMD (mgHA/cm^3^)** | 306.32 | 69.97 | 301.80 | 72.76 | 306.06 | 69.96 | 298.14 | 67.51 | 289.03 | 65.07 | 293.53 | 64.69 | 288.27 | 64.99 | 289.98 | 71.19 | 301.60 | 65.39 | 305.10 | 61.83 | 303.01 | 61.59 | 298.84 | 60.82 | 285.11 | 48.18 | 283.40 | 54.55 | 284.39 | 56.30 | 284.09 | 55.59 |
| **Trabecular volumetric**  **BMD (mgHA/cm^3^)** | 153.06 | 39.30 | 147.97 | 36.29 | 149.16 | 36.40 | 141.91 | 33.15 | 147.33 | 40.18 | 150.59 | 41.02 | 148.73 | 41.59 | 149.42 | 43.66 | 138.74 | 45.27 | 138.41 | 40.64 | 138.18 | 41.60 | 136.61 | 41.17 | 148.83 | 33.89 | 147.16 | 41.46 | 147.43 | 41.98 | 146.65 | 42.15 |
| **BV/TV (%)** | 0.13 | 0.03 | 0.12 | 0.03 | 0.12 | 0.03 | 0.12 | 0.03 | 0.12 | 0.03 | 0.13 | 0.03 | 0.12 | 0.03 | 0.12 | 0.04 | 0.12 | 0.04 | 0.12 | 0.03 | 0.12 | 0.03 | 0.11 | 0.03 | 0.12 | 0.03 | 0.12 | 0.03 | 0.12 | 0.04 | 0.12 | 0.04 |
| **Trabecular inhomogeneity (Tb.1/N.SD; mm)** | 0.24 | 0.11 | 0.25 | 0.12 | 0.26 | 0.13 | 0.27 | 0.13 | 0.33 | 0.25 | 0.33 | 0.26 | 0.31 | 0.25 | 0.31 | 0.24 | 0.36 | 0.26 | 0.38 | 0.30 | 0.36 | 0.29 | 0.38 | 0.30 | 0.27 | 0.13 | 0.30 | 0.17 | 0.31 | 0.20 | 0.31 | 0.19 |
| **Cortical area (mm^2^)** | 51.23 | 8.35 | 51.11 | 7.52 | 51.30 | 7.70 | 51.90 | 7.58 | 47.31 | 7.93 | 48.22 | 8.37 | 47.70 | 8.39 | 49.81 | 17.74 | 50.33 | 8.01 | 51.22 | 8.17 | 51.03 | 7.80 | 50.47 | 8.25 | 46.30 | 6.44 | 45.95 | 6.40 | 46.36 | 6.70 | 46.18 | 6.41 |
| **Cortical vBMD (mgHA/cm^3^)** | 859.76 | 59.52 | 859.03 | 62.70 | 864.45 | 62.07 | 860.67 | 62.29 | 838.48 | 52.77 | 838.98 | 52.84 | 832.96 | 51.20 | 825.59 | 80.84 | 879.37 | 62.30 | 883.09 | 59.47 | 877.71 | 56.92 | 876.57 | 58.34 | 838.91 | 45.61 | 837.02 | 49.81 | 836.76 | 48.24 | 837.40 | 47.14 |
| **Cortical perimeter (mm)** | 68.09 | 6.72 | 68.11 | 6.66 | 67.62 | 6.86 | 68.27 | 7.01 | 69.27 | 12.71 | 67.06 | 5.90 | 67.16 | 6.02 | 66.87 | 5.78 | 66.00 | 4.83 | 66.02 | 4.68 | 65.74 | 4.78 | 65.79 | 4.66 | 67.90 | 5.23 | 67.32 | 5.19 | 67.25 | 5.02 | 66.92 | 5.00 |
| **Cortical thickness (mm)** | 0.84 | 0.15 | 0.83 | 0.15 | 0.84 | 0.15 | 0.84 | 0.15 | 0.79 | 0.17 | 0.81 | 0.16 | 0.80 | 0.16 | 0.82 | 0.23 | 0.85 | 0.15 | 0.86 | 0.16 | 0.86 | 0.16 | 0.85 | 0.16 | 0.77 | 0.13 | 0.77 | 0.12 | 0.77 | 0.13 | 0.78 | 0.13 |
| **Cortical porosity (%)** | 0.02 | 0.01 | 0.02 | 0.01 | 0.02 | 0.01 | 0.02 | 0.01 | 0.03 | 0.01 | 0.03 | 0.01 | 0.03 | 0.01 | 0.05 | 0.10 | 0.02 | 0.01 | 0.02 | 0.01 | 0.02 | 0.01 | 0.02 | 0.01 | 0.02 | 0.01 | 0.02 | 0.01 | 0.02 | 0.01 | 0.02 | 0.01 |
| **Radius ultimate load (N)** | -2909.28 | 525.90 | -2829.55 | 492.52 | -2860.76 | 486.30 | -2892.36 | 537.87 | -2828.00 | 570.99 | -2848.34 | 559.54 | -2740.08 | 553.43 | -2792.04 | 599.15 | -2957.39 | 548.81 | -2908.60 | 652.85 | -2938.53 | 540.81 | -2866.34 | 546.86 | -2817.38 | 528.03 | -2791.71 | 591.60 | -2744.81 | 544.35 | -2769.88 | 517.82 |
| **Trabecular load vs whole**  **bone load (distal)** | 0.43 | 0.06 | 0.41 | 0.07 | 0.40 | 0.06 | 0.40 | 0.08 | 0.45 | 0.08 | 0.45 | 0.09 | 0.44 | 0.07 | 0.46 | 0.08 | 0.43 | 0.10 | 0.41 | 0.10 | 0.41 | 0.09 | 0.41 | 0.10 | 0.47 | 0.09 | 0.48 | 0.10 | 0.45 | 0.10 | 0.47 | 0.09 |
| **Trabecular load vs whole bone load (proximal)** | 0.14 | 0.04 | 0.13 | 0.04 | 0.13 | 0.04 | 0.13 | 0.04 | 0.17 | 0.06 | 0.17 | 0.06 | 0.17 | 0.06 | 0.17 | 0.05 | 0.14 | 0.06 | 0.13 | 0.06 | 0.14 | 0.05 | 0.14 | 0.05 | 0.17 | 0.07 | 0.18 | 0.07 | 0.17 | 0.07 | 0.17 | 0.07 |
| **Trabecular Von Mises stress (MPa)** | 41.24 | 5.85 | 39.81 | 6.28 | 40.03 | 5.95 | 39.79 | 5.96 | 43.17 | 6.51 | 43.66 | 5.90 | 41.45 | 6.76 | 43.48 | 6.81 | 43.82 | 7.76 | 42.93 | 8.78 | 42.84 | 7.27 | 42.27 | 7.71 | 43.29 | 6.48 | 43.73 | 7.97 | 42.23 | 7.31 | 43.61 | 7.30 |
| **Cortical Von Mises stress(MPa)** | 78.99 | 3.34 | 78.15 | 3.46 | 79.00 | 3.10 | 78.81 | 4.00 | 78.88 | 4.13 | 78.82 | 3.99 | 77.84 | 4.19 | 77.69 | 5.58 | 80.98 | 3.27 | 79.21 | 11.22 | 80.96 | 3.29 | 80.13 | 3.62 | 78.50 | 3.49 | 78.48 | 3.61 | 78.33 | 3.46 | 78.55 | 3.74 |

**SUPPLEMENTAL DATA 11:** Descriptive statistics for serum parameters with age groups (≤64&>64)

|  | **Group** | | | | | | | | | | | | | | | | | | | | | | | | | | | | | | | |
| --- | --- | --- | --- | --- | --- | --- | --- | --- | --- | --- | --- | --- | --- | --- | --- | --- | --- | --- | --- | --- | --- | --- | --- | --- | --- | --- | --- | --- | --- | --- | --- | --- |
|  | **Control** | | | | | | | | | | | | | | | | **Vibration** | | | | | | | | | | | | | | | |
|  | **Age groups** | | | | | | | | | | | | | | | | **Age groups** | | | | | | | | | | | | | | | |
|  | **Less than ≤64** | | | | | | | | **Greater than 64** | | | | | | | | **Less than ≤64** | | | | | | | | **Greater than 64** | | | | | | | |
|  | **Month** | | | | | | | | **Month** | | | | | | | | **Month** | | | | | | | | **Month** | | | | | | | |
|  | **Baseline** | | **Month 6** | | **Month 12** | | **Month 18** | | **Baseline** | | **Month 6** | | **Month 12** | | **Month 18** | | **Baseline** | | **Month 6** | | **Month 12** | | **Month 18** | | **Baseline** | | **Month 6** | | **Month 12** | | **Month 18** | |
|  | **Mean** | **SD** | **Mean** | **SD** | **Mean** | **SD** | **Mean** | **SD** | **Mean** | **SD** | **Mean** | **SD** | **Mean** | **SD** | **Mean** | **SD** | **Mean** | **SD** | **Mean** | **SD** | **Mean** | **SD** | **Mean** | **SD** | **Mean** | **SD** | **Mean** | **SD** | **Mean** | **SD** | **Mean** | **SD** |
| **P1NP (µg/L)** | 62.12 | 24.76 | 62.49 | 25.30 | 56.64 | 22.56 | 55.09 | 22.87 | 52.24 | 22.81 | 52.85 | 20.47 | 54.83 | 22.96 | 55.18 | 22.63 | 56.69 | 19.04 | 55.35 | 20.04 | 60.21 | 19.64 | 55.51 | 19.52 | 55.36 | 17.55 | 55.46 | 18.29 | 54.40 | 19.08 | 52.99 | 16.29 |
| **CTX (pmol/L)** | 4782.31 | 2735.51 | 5127.47 | 2900.64 | 4571.64 | 2436.41 | 4408.54 | 2220.12 | 3784.79 | 2095.48 | 3994.41 | 2160.93 | 4004.40 | 2157.95 | 4453.19 | 2488.44 | 4544.17 | 1947.04 | 4826.54 | 2207.42 | 4989.14 | 2219.55 | 5029.45 | 2234.78 | 4007.67 | 1621.42 | 4271.53 | 2032.06 | 4697.90 | 1762.57 | 5225.33 | 2139.42 |
| **BAP (µg/L)** | 12.38 | 4.31 | 13.48 | 4.42 | 13.79 | 4.20 | 14.36 | 3.53 | 12.17 | 4.05 | 13.57 | 4.51 | 14.94 | 5.56 | 16.17 | 6.28 | 11.58 | 3.49 | 12.59 | 3.23 | 13.74 | 3.81 | 13.94 | 4.22 | 11.92 | 3.80 | 13.25 | 4.19 | 14.68 | 4.62 | 15.11 | 4.40 |
| **Sclerostin (ng/mL)** | 0.57 | 0.09 | 0.56 | 0.10 | 0.55 | 0.09 | 0.57 | 0.10 | 0.68 | 0.16 | 0.69 | 0.17 | 0.67 | 0.17 | 0.67 | 0.17 | 0.57 | 0.14 | 0.57 | 0.14 | 0.58 | 0.14 | 0.59 | 0.19 | 0.63 | 0.15 | 0.60 | 0.14 | 0.62 | 0.15 | 0.61 | 0.16 |
